# Essential mineral elements in roe deer: Associations with parasites and immune phenotypes in two contrasting populations

**DOI:** 10.1002/ece3.11613

**Published:** 2024-10-29

**Authors:** Léa Bariod, Sonia Saïd, Clément Calenge, Renaud Scheifler, Clémentine Fritsch, Carole Peroz, Slimania Benabed, Hervé Bidault, Stéphane Chabot, François Débias, Jeanne Duhayer, Sylvia Pardonnet, Marie‐Thérèse Poirel, Paul Revelli, Pauline Vuarin, Gilles Bourgoin

**Affiliations:** ^1^ Université de Lyon, VetAgro Sup – Campus Vétérinaire de Lyon, Laboratoire de Parasitologie Vétérinaire Marcy‐L'Etoile France; ^2^ Université de Lyon, Université Lyon 1, CNRS, UMR 5558, Laboratoire de Biométrie et Biologie Évolutive Villeurbanne France; ^3^ Office Français de la Biodiversité, Direction de la Recherche et de l'Appui Scientifique, Service Conservation et Gestion des Espèces à Enjeux, «Montfort» Birieux France; ^4^ Office Français de la Biodiversité, Direction de la Surveillance, de l'Évaluation et des Données – Unité données et appui méthodologique Le Perray en Yvelines France; ^5^ Université de Franche‐Comté, UMR CNRS 6249, Laboratoire Chrono‐environnement Besançon France; ^6^ Office Français de la Biodiversité, Direction de la Recherche et de l'Appui Scientifique, Service Conservation et Gestion Durable des Espèces Exploitées Chizé France; ^7^ Office Français de la Biodiversité, Direction de la Recherche et de l'Appui Scientifique, Service Conservation et Gestion Durable des Espèces Exploitées Trois‐Fontaines‐L'Abbaye France

**Keywords:** *Capreolus capreolus*, environment, immunity, juvenile, trace elements, wild ungulate

## Abstract

Low levels of essential mineral elements such as cobalt, copper, and iron, in organisms reduce immune function, increasing the chances of parasitic infection. This phenomenon has been demonstrated widely in domestic animals but rarely in wildlife. In this study, we used data from 7‐ to 9‐month‐old roe deer (*Capreolus capreolus*), living in two different populations facing contrasting environmental conditions (Trois‐Fontaines and Chizé), to investigate whether the parasitic and immunological statuses could be related to essential element status. Between 2016 and 2019, we collected feces to measure parasite burdens (gastrointestinal and pulmonary nematodes), blood to measure immunological parameters (globulins and white blood cells), and hair to assess the concentration of 11 essential elements (calcium [Ca], chromium [Cr], cobalt [Co], copper [Cu], iron [Fe], magnesium [Mg], manganese [Mn], potassium [K], molybdenum [Mo], selenium [Se], and zinc [Zn]). The results showed first heterogeneity in the individual phenotypes of the two populations. Roe deer with low body mass had high concentrations of all the essential elements (in particular, Ca, Fe, Cu, K, and Mn), a high parasitic burden, and high concentrations of globulins. An association between high concentrations of essential elements and a high parasite burden was found at the two study sites despite markedly different environmental conditions. A relationship between essential element concentrations and immune parameters was also detected, with more basophils and globulins being associated with high concentrations of essential trace elements (i.e., Ca, Fe, Cu, and, to a lesser extent, Se, Cr, and Zn). These results suggest that young individuals with low body mass and high parasitism may select feeding resources rich in mineral elements, which may improve their ability to control the infestation and/or mitigate the negative consequences of parasites by maintaining immune system functions.

## Graphical abstract

Essential trace elements are known to be necessary in an individual's life to perform various metabolic functions, for example, immunity and defense against parasites. For the first time, this study brings a new vision of the trace element‐parasite‐immunity relationships in two populations of roe deer living in contrasting habitats.

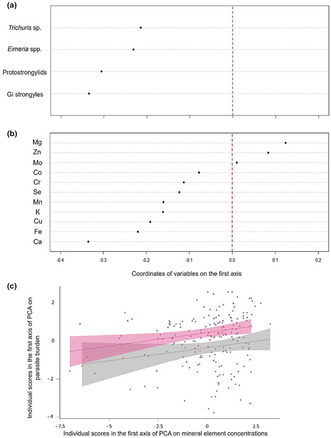

## 1 INTRODUCTION

Parasites are considered natural stressors for hosts due to their influence on metabolism and behavior (Budischak et al., 2018; Joly et al., 2020). The negative effects of parasites on the host can vary depending on parasite virulence (i.e., mode of transmission, burden, and species diversity; Bordes & Morand, 2011; Budischak et al., 2018) and host tolerance and resistance (i.e., the host's ability to mitigate parasite‐inflicted damage or to reduce infection) (Råberg et al., 2009). Heterogeneity of parasitic infestation between individuals is common, with some individuals within the same population being more heavily parasitized than others (Woolhouse et al., 1997). Young individuals, in their first year, and senescent individuals (i.e., from the time there is a decline in reproductive success and survival) are often more infested and sensitive to parasites than adults are (Body et al., 2011; Bourgoin et al., 2021; Cheynel et al., 2017). This could be explained by the developing immune system in young individuals (Coop & Kyriazakis, 2001) and by immunosenescence in senescent individuals (inflammaging; Cheynel et al., 2017). Additionally, males are often more parasitized than females are, likely due to physiological and ecological differences between sexes, particularly the costs associated with sexual selection in males (Body et al., 2011; Forbes, 2007; Jégo et al., 2014; Turner & Getz, 2010).

The nutritional status and body condition of hosts also play essential roles in the establishment and survival of parasite populations (Beldomenico & Begon, 2010; Parkins & Holmes, 1989). Previous studies have shown that individuals with poorer body conditions often have greater parasite burdens (e.g., Aleuy et al., 2018; Davidson et al., 2015), which may result from the effects of the infection (Begon & Townsend, 2020; Beldomenico et al., 2008) or from the poor environmental conditions where those individuals live (i.e., low quantity or poor quality of food). Parasites, such as helminths and lungworms, can cause food intake depression in hosts (Fox, 1997), alter their metabolism (e.g., inhibiting the ability of the host to store or use lipids and proteins; Coop & Holmes, 1996; Gulland, 1992), and lead to a reduction in mass gain (Corrigall et al., 1982). For example, parasitic infection can induce protein deficiency by increasing the demand for amino acids in the digestive tract while reducing nutrient intake through appetite depression (as shown in sheep, Sykes & Coop, 2001). On the other hand, hosts living in habitats with low quantities and quality of food resources may have higher parasitism due to a higher risk of encountering parasites. For instance, when resources are scarce, animals might aggregate where food patches remain, providing opportunities for parasite transmission (Becker et al., 2015). Many animals can also expand their home range to find resources (Harestad & Bunnel, 1979; Relyea et al., 2000), which could increase the risk of encountering parasites (Ostfeld et al., 2005). Individuals living in harsh environmental conditions with food scarcity, poor nutritional quality, and high host density may be prone to parasite infection as they may have less resources and energy to gain mass and maintain immune functions necessary to fight infection. Immune activation is costly in terms of energy and protein, and food deprivation can lead to a reduction in specific cellular and humoral immune responses, particularly to fight against parasites (Cotter & Al Shareefi, 2022). For example, in insects, a study by Banville et al. (2012) showed that food deprivation in *Galleria mellonella* larvae resulted in reduced cellular and immune responses and increased susceptibility to infections. In mammals such as sheep, dietary protein supplementation results in a higher expulsion rate of *Trichostrongylus colubriformis* larvae (van Houtert et al., 1995), revealing the importance of nutrition in the expression of immunity. However, not all immune effectors react in the same way to dietary manipulation, and will depend on the needs of the host to maintain other functions (Cotter & Al Shareefi, 2022). Moreover, increased immune responses are not always beneficial for individuals and can lead to immunopathology; for example, autoinflammatory disease manifested by tissue aggression linked to excessive activation of innate immunity (Sibilia, 2007), therefore, it is important to be careful in interpreting the results of studies.

Veterinary research is particularly interested in the consequences of nutrition and parasitism in farmed animals, particularly for economic and health reasons (Asano et al., 2002; Coop & Holmes, 1996; Rosendahl et al., 2022; Sordillo, 2016). Studies on this subject have considered these interactions within a framework that considers the physiology of the infection and the distribution of nutritive resources such as glucids, proteins, or even mineral elements to various bodily functions, including defense against pathogens (Coop & Kyriazakis, 1999). Most of the understanding of the influence of parasites, primarily gastrointestinal nematodes, on metabolism comes from experimental infections in sheep (Cotter & Al Shareefi, 2022). Mineral elements have received particular attention because they include micro‐ and macronutrients, which are essential for all living organisms (e.g., Cu, Se, Zn; Paul & Dey, 2015) and may also influence host sensitivity to parasites (Atiba et al., 2020; Čobanová et al., 2020; McClure, 2003; McClure et al., 1999). As mineral elements work globally as cofactors for various enzymes notably involved in immune function (Ahmad et al., 2020), their inadequate intake can have major impacts on an individual's physiology and health, affecting the immune response. In fact, these elements are involved in processes such as the maintenance of physical barriers (i.e., skin and mucous membranes), the differentiation and proliferation of cells, and inflammation and antioxidant protection (i.e., protein secretion) (Hughes & Kelly, 2006; McClure, 2008; Paul & Dey, 2015). In livestock, several micronutrients, such as Co, Cr, Cu, Fe, Mn, Mo, Se, and Zn, are likely to be positively involved in gut immunity via physiological roles in regulating or triggering immunity (McClure, 2008). For instance, zinc deficiencies have been shown to cause atrophy of lymphoid tissues, leading to decreased numbers of immune cells and functional defects during antigen‐specific responses (Scott & Koski, 2000) and increasing the sensitivity of individuals suffering from such disequilibrium to parasitic infections. Macroelements, such as calcium, magnesium, and potassium, are involved mainly, but not exclusively, in the structure of molecules as well as in enzymatic systems, which also partly explains the need for their substantial intake (Lippert, 2020). Therefore, mineral elements, either in minimal (micro) or larger (macro) quantities, are essential for ensuring optimal functioning of the organism, particularly in response to parasites. Most of the literature on this subject, however, addresses domestic animals (Ahmad et al., 2020; Atiba et al., 2020; Čobanová et al., 2020; McClure, 2008; Paul & Dey, 2015). To our knowledge, only two studies on wild ungulates (i.e., *Odocoileus hemionus*, Myers et al., 2015; *Rangifer tarandus caribou*, Rioux et al., 2022) have focused on parasite burden and mineral elements, although they did not highlight a general trend between these parameters. Thus, the relationships between the parasitic/immunological statuses and the essential mineral element status in wild systems remain to be determined.

In the present study, we focused on a wild ungulate, the roe deer (*Capreolus capreolus*), which is a highly sedentary species (Andersen et al., 1998; Gaudry et al., 2018; Saïd et al., 2009). Roe deer may be infected by multiple parasite species at any one time this can include gastrointestinal helminths and lungworms. Both of which can have a negative impact on nutritional status and growth rate (Pato et al., 2013; Segonds‐Pichon et al., 2000; Zaffaroni et al., 1997). Young roe deer are more susceptible to parasitic infection (Body et al., 2011) due to their developing immune system (Coop & Kyriazakis, 2001) and exhibit greater heterogeneity in terms of infestation levels than adults. In addition, as they have specific nutritional requirements for growth (Hewison et al., 2002), young roe deer are more likely to be dependent on high‐quality food resources (e.g., with lots of nutrients and mineral elements). Thus, young roe deer are a relevant model for understanding the links between essential element concentrations, parasite burdens, and immune phenotypes.

**FIGURE 1:**
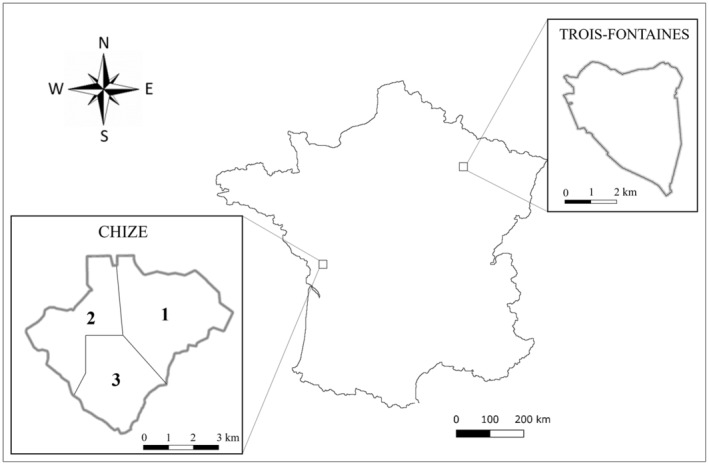
The geographical location of the two populations in France. On the left: a spatial representation of the Chizé site with the three distinct vegetation areas: (1) oak‐hornbeam, (2) oak‐Montpellier maple, and (3) beech. On the right: spatial representation of the Trois‐Fontaines site.

We studied 7–9‐month‐old roe deer caught during winter (January–March) between 2016 and 2019 in two populations exhibiting markedly different ecological contexts (Trois‐Fontaines and Chizé). Indeed, while Trois‐Fontaines is a rich forest with abundant resources overall, Chizé is a poorer forest with limited resources stratified into three habitats (Figure 1), contrasting in forest structure and food resource quality (Gaillard et al., 1993; Pettorelli et al., 2005; Saïd et al., 2005; Saïd & Servanty, 2005; Gaudry et al., 2018). To carry out this study, we used data from samples taken during roe deer captures: parasite burden measured in feces, immune phenotype measured in blood, and mineral element status measured in hair. Generally, mineral element levels are quantified in storage organs (liver and kidneys) or in blood (Jutha et al., 2022) to understand the current condition of an individual. However, hair has been recognized as a good matrix for assessing an organism's long‐term exposure to mineral elements (Draghi et al., 2023; Mosbacher et al., 2022; Patra et al., 2006; Roug et al., 2015). Hair is composed of three distinct layers that each play a role in the absorption of mineral elements: the cuticle, which contains sulfur and can absorb elements (Noguchi et al., 2012); and the medulla and cortex, which contain little or no sulfur but are embedded in pigment granules and can selectively bind mineral elements (Tobin, 2008). Due to these characteristics, using hair to monitor mineral element status in a natural population is a reliable method. In roe deer, the coat is renewed twice a year: the winter molt begins in September and lasts approximately 2 months, while the spring molt begins in May and lasts 2 months (Johnson & Hornby, 1975). Thus, hair samples collected during winter captures reflect mineral elements incorporated during the period of hair growth in the autumn of the previous year, that is, the period when food resources are very important for fawn growth, providing an indicator of an individual's condition at the time of hair formation (Katz & Chatt, 1994).

Therefore, using concentrations in hair would allow us to determine the importance of the essential mineral elements that are ingested during this critical period for body condition and, consequently, for immune defense and the control of parasitism. Indeed, depending on the nutritional intake in autumn and early winter, an individual with higher concentrations of essential mineral elements would ensure adequate immune function to eliminate parasites (McClure, 2008). In such a situation, no or low parasite burden and high concentrations of certain immunological parameters are expected (e.g., basophils, Abbas et al., 2023; Obata‐Ninomiya et al., 2020). Currently, a recent study described the concentrations of 22 mineral elements present in roe deer (Herrada et al., 2024), but no link has been established between these concentrations and health parameters in individuals, for example, parasitic burden. Thus, we predicted a negative association of parasite burden (P1) and a positive association of immunological parameters (P2) with mineral element concentrations. Moreover, body mass reflects the nutritional status of individuals and, indirectly, its consequences for the immune response and susceptibility to parasites. Hence, an individual with a low body mass, due to a low intake of good quality food resources (i.e., low quantity or poor quality of food, loss of appetite due to parasites), was expected to have lower concentrations of mineral elements, poorer immune defenses, and a greater parasitic burden compared to an individual with a larger body mass (P3). Finally, the quality of the habitat, in particular the quantity and quality of food resources, determines nutrient availability to hosts for their immune systems and the control of parasites. We thus expected to find lower mineral element concentrations and greater variability in phenotypic profiles, that is, in terms of mineral status, parasite burden, and immune status, in individuals living in areas with poorer resource quality, typical of Chizé compared to Trois‐Fontaines (P4a), but also inside the Chizé site, in the area of lower quality (Figure 1) (P4b).

## 2 MATERIALS AND METHODS

### 2.1 Ethical considerations

Roe deer captures and experimental procedures were in line with the French Environmental Code (Art. R421‐15 to 421‐31 and R422‐92 to 422‐94‐1). The study was approved by the authorities (French Ministry of Environment) and performed in accordance with the conditions detailed in the specific accreditation delivered to the “Office Français de la Biodiversité” by the “Préfecture de Paris” (agreement no. 2009–014, no. 2013–118, no. 2019‐02‐19‐003). The experiments were carried out by minimizing the stress and handling time of the animals (limited to 15 min) and by ensuring their welfare, thus respecting the European and French laws defined for the ethical use of animals in research.

### 2.2 Study populations

Roe deer from two French populations at two enclosed study sites, namely, “Territoire d'Étude et d'Expérimentation of Trois‐Fontaines” (hereafter referred to as TF) and “Réserve Biologique Intégrale of Chizé” (hereafter referred to as CH), were studied between 2016 and 2019 (Figure 1). During this period, the density of roe deer at TF oscillated between 12 and 15 individuals/100 ha, and that at CH oscillated between 13 and 18 individuals/100 ha (numbers estimated by capture–mark–recapture models). These two study sites differ in terms of habitat quality, that is, soil fertility, vegetation structure, and the quantity and quality of resources available for roe deer (Gaudry et al., 2018; Saïd et al., 2009), which could provide resources with different nutritional qualities and influence the concentrations of mineral elements ingested by individuals. TF is located in northeastern France (48°38′ N, 4°54′ E), where the climate is continental and characterized by cold winters and hot but not dry summers. This is an enclosed forest of 1360 ha with an overstory dominated by oak (*Quercus* spp.) and beech (*Fagus sylvatica*), while the coppice is dominated by hornbeam (*Carpinus betulus*). The high productivity and rich soil (i.e., deep, fertile) at the site provide a high‐quality habitat for roe deer (Pettorelli et al., 2006). CH is located in western France (46°50′ N, 0°25′ E), where the climate is temperate oceanic with Mediterranean influences. This site is characterized by poor soil quality (i.e., shallow, stony) and low productivity, offering poorer habitat quality for roe deer (Pettorelli et al., 2006). In this enclosed forest of 2614 ha, three contrasted habitats in a timber stand and coppices were distinguished: the northeastern area (Area 1), covered by oak forest with a coppice dominated by hornbeam; the northwestern area (Area 2), covered by oak forest with Montpellier maple (*Acer monspessulanum*) coppice; and the southern area (Area 3), represented by beech forest. Moreover, plants in Area 1 have a greater nitrogen content than those in the other areas, especially Area 3 (Pettorelli et al., 2001), which makes oak and hornbeam forests richer habitats and of better quality for roe deer than the other types of forests.

The two populations have been monitored using capture–mark–recapture methods since 1976 (TF) and 1978 (CH) (Gaillard et al., 1993). Driving nets are used each year between December and March to catch individuals (10–12 days of capture per year). In this study, we considered only 7–9‐month‐old roe deer captured between 2016 and 2019 (for each year, data were collected between January and March; see Table S1A). Age was estimated based on dentition (two premolars only, large incisors; Maublanc et al., 1991; Delorme et al., 2007) or on whether the individual had already been captured as fawn the year before and thus identified (i.e., marked by an ear tag). Once an individual was captured, its sex and body mass (to the nearest 50 g) were recorded. On average, the roe deer from the TF were heavier (mean ± SD; 15.39 ± 2.49 kg) than those from the CH were (area 1: 12.91 ± 2.32 kg; area 2: 11.46 ± 2.03 kg; area 3: 12.37 ± 2.44 kg) (see Figure S1). In addition, various biological samples (feces, blood, and hair) were collected. In total, we studied 233 roe deer (111 at TF and 122 at CH). However, it was not always possible to collect all the biological samples from each individual or to obtain enough samples for analysis, leading to unbalanced sample sizes (mineral element concentrations, *n* = 233 [111 in TF, 122 in CH]; parasite burden, *n* = 190 [87 in TF, 103 in CH]; and immunological parameters, *n* = 105 [54 in TF, 51 in CH]).

### 2.3 Roe deer data

#### 2.3.1 Mineral element concentrations in hair

Mineral element concentrations were measured at the Chrono‐Environnement Laboratory (Besançon, France) in hair collected from the white patch around the rump of the captured roe deer. A handful of hair was removed, the bulb was carefully harvested, and the hair was stored in paper envelopes at room temperature. Using hair from the same region of the body helps to avoid measurement bias, especially for the dosage of minerals (Combs, 1987). The hair samples weighed 117 ± 32 mg on average.

Only mineral elements considered essential [i.e., calcium (Ca), cobalt (Co), chromium (Cr), copper (Cu), iron (Fe), potassium (K), magnesium (Mg), manganese (Mn), molybdenum (Mo), selenium (Se), and zinc (Zn)] for metabolism were analyzed in this study (Underwood, 2012). The term “mineral elements” will therefore refer only to these essential elements. First, the hair samples were washed as advised by Peakall and Burger (2003) to remove potential external contamination so that only the mineral elements accumulated in (and not deposited on) the hair were measured. Washing involved six successive baths of 5 min in an ultrasonic cleaner device (140/280 W, 37 kHz, Fisher Scientific, S‐LINE 4.25 L), alternatively using three baths of acetone and three baths of ultrapure water (UP water, Elga, 18.2 MΩ cm). The samples were subsequently dried at 60°C, digested with nitric acid, and diluted by adding ultrapure water prior to analysis. Concentrations of Ca, K, and Mg were measured by inductively coupled plasma–atomic emission spectrometry (ICP–AES; Thermo Fisher Scientific iCAP 6000), and concentrations of Co, Cr, Cu, Fe, Mn, Mo, Se, and Zn were measured by inductively coupled plasma–mass spectrometry (ICP–MS; Thermo Fisher Scientific XSeries 2). A certified reference material (Tabac obtl5) was also analyzed to validate the results. The reproducibility of the measurements was surveyed by checking relative standard deviation values and using internal standards, and quality control using control solutions and blanks to check for the absence of drift was regularly conducted during the measurements.

#### 2.3.2 Parasitological data

The feces of each captured roe deer were collected manually from the rectum and placed in plastic bags, from which the air was removed before sealing. In the field, bags were stored in an insulated box to avoid exposure to low temperatures. The samples were then either directly transported or mailed to the parasitology laboratory of VetAgro Sup (Marcy‐l'Etoile, France) within 24–48 h following fecal collection. Four groups of parasite species commonly found in roe deer from our study populations (Body et al., 2011; Cheynel et al., 2017) were investigated: gastrointestinal nematodes (strongyles [hereafter GI strongyles] and *Trichuris* sp.), *Eimeria* spp. (protozoan), and pulmonary nematodes (protostrongylids). On receipt of the fecal samples, two different techniques were used to measure the number of parasite propagules per gram of feces: (i) the modified McMaster protocol with a solution of zinc sulfate (ZnSO_4_, s.g. = 1.36) (Raynaud et al., 1970) and a theoretical sensitivity of 15 eggs per gram (EPG; for gastrointestinal nematodes and *Trichuris* sp.) or oocysts per gram (OPG; for *Eimeria* spp.) of feces; and (ii) the Baermann fecal technique (Baermann, 1917) for first‐stage larvae of pulmonary nematodes (larvae/g of feces, LPG). On average, 3.63 ± 1.47 g of feces was used to carry out the McMaster protocol, and 2.43 ± 1.67 g of feces was used to carry out the Baermann technique.

These measures of fecal excretion of parasite propagules (FEPP) can be considered proxies for parasitic infection of the host. However, FEPP provides only an overview of the parasite burden of an individual and cannot be measured directly. Several factors might influence the production of propagules by parasites and the results of a fecal egg count, such as the immune response of the host, the diversity of parasite species (variability of fecundity among species, including species of the same group of parasites such as gastrointestinal strongyles), the density‐dependent production of propagules (production of propagules decreases as parasite burden increases), the temporal variation in propagule production, and the heterogeneous distribution of propagules in feces (e.g., Cabaret et al., 1998; Denwood et al., 2012; Nielsen, 2022). In this study, we use the term “parasite burden” to refer to these measurements, keeping in mind that this is an overview of what is found in deer feces, not in their bodies.

#### 2.3.3 Immunological parameters

Blood samples (EDTA and dry tubes) were collected from the jugular vein (with a maximum volume of 1 mL/kg) of each individual. Whole blood was preserved on EDTA at 4°C for white blood cell counts (analyzed within 52 h of sampling), and the serum, extracted by centrifugation from dry tubes immediately upon blood collection, was stored at −20°C for functional measures of activity (i.e., humoral immunity). Hematological assays were performed at the Biochemical and Endocrinological Laboratory of VetAgro‐Sup.

Humoral immunity was assessed by measuring the circulating levels of natural antibodies and complement‐mediated cell lysis activity following hemagglutination‐hemolysis. The total protein content (in g/L) was assessed by refractometry followed by automatic agarose gel electrophoresis (HYDRASYS, Sebia, Evry, France), which separates the albumin and globulin fractions. In our study, we focused on gamma‐ and beta‐globulin levels (mg/mL), which encompass some of the components involved in defense against parasites and should reflect the antiparasitic response. Both are involved in the inflammatory response and reflect the inflammatory condition of the animal during the few days preceding capture. The gamma‐globulin fraction is mainly composed of antibodies (IgG, IgA, and IgE) and the beta‐globulin fraction of IgM and IgA, as well as other proteins involved in innate immunity. In addition, we assessed innate cellular immunity by counting white blood cells (WBCs, in 10^3^ cells/mL), which is considered a proxy for the allocation to immune defenses, using a Konelab 30i automaton (Fisher Thermo Scientific, Cergy‐Pontoise, France). The proportions of each WBC type were quantified under a microscope (×1000) by counting the first 100 WBCs on blood smears previously stained with May‐Grünwald and Giemsa solutions (Houwen, 2001). Two innate cell types were analyzed here: basophils, which play a role in the defense against macroparasites (e.g., parasitic helminths and ticks), and eosinophils, which are implicated in the defense against internal parasites and the inflammatory response (Mitre & Nutman, 2006). The parameters mentioned above reflect the immunological status from the few weeks before capture to the date of capture. Basophils and eosinophils remain in blood for short periods only before they home to tissues or organs, but their number increases in the case of parasitic infection, and their count reflects an animal's parasite burden. However, immunoglobulins have longer half‐lives (several weeks, depending on the isotype).

### 2.4 Data analysis

All the statistical procedures were performed using R version 4.0.4 (R Core Team, 2021) and the package “ade4” (Thioulouse et al., 2018) to perform multivariate analyses. The significance threshold was set at the *α* = 0.05 level. Mineral element concentrations, parasite burdens, and immunological parameters (i.e., beta‐globulin and gamma‐globulin) were asymmetrically distributed, leading to the risk that a small number of animals characterized by high values of these variables would strongly support the results of our analysis. We therefore log‐transformed these variables to limit such influences. Basophil and eosinophil counts were analyzed as prevalence (presence/absence) because of the low values and variability of concentration of each cell type in the blood samples of the studied roe deer (basophils: prevalence = 0.26 [*n* = 27/105]; median concentration: 0 g/L; eosinophils: prevalence = 0.48 [*n* = 50/105]; median concentration: 0.076 g/L).

We studied several mineral elements, immunological parameters, and parasites, and we wanted to assess the relationships between these sets of variables. We were not interested in the specific relationship between, for example, one mineral element and one parasite but rather between mineral elements taken as a whole (i.e., mineral status) and parasites taken as a whole (i.e., parasite burden). We therefore used a statistical methodology tailored for this task, that is, factorial analysis methods. These methods search for synthetic variables, summarizing each set of variables. The weight of each variable of a given set in this synthetic variable was a coefficient that measured its importance in the definition of the synthesis. We first carried out a principal component analysis (PCA) on the mineral element concentrations and parasite burdens and a Hill & Smith analysis (HSA) on the immunological parameters to identify the correlation patterns within each set of variables. These analyses allow us to obtain the most relevant summary possible of the initial data, that is, by looking at the synthetic variable(s) most correlated with all these parameters. To identify the number of components to interpret in a given PCA or HSA, we used a parallel analysis (package “paran”; Dinno, 2009, 2022) developed by Horn in 1965 (Horn, 1965). We then carried out several coinertia analyses to explore the association patterns between the different datasets. Coinertia analysis is especially suitable for exploring the covariance structure between tables paired by rows (Dolédec & Chessel, 2006; Thioulouse et al., 2018). The coinertia analysis of two tables (for example, the table of mineral element concentration and the table of parasite burden) first involved finding two linear combinations of the columns of the first and second tables, respectively (called factorial axes), that are characterized by the largest possible covariance. These two linear combinations describe the costructure between the two tables. Then, this method searches for two more linear combinations of the columns of the first and second tables with the largest possible covariance under the constraint that these combinations must be uncorrelated with the first set of factorial axes, and so on. These two linear combinations can be used to calculate two sets of scores for the animals in the dataset (and the mean of these two scores will be further called “individual scores”) and to describe the costructure between the two tables. Thus, we performed a coinertia analysis between mineral element concentrations and each other table (parasite burden [Prediction 1] and immunological parameters [Prediction 2]) to relate mineral element profiles defined by the PCA with each set of explanatory variables in the two other tables. Given the differences in sample sizes between the mineral element data (*n* = 233) and the other tables (*n* = 190 for parasite burden, *n* = 105 for immunological parameters), we considered only individuals with data on both mineral elements and parasite burden/immunological parameters to perform the coinertia analyses. We also computed the RV coefficient (a multivariate generalization of the Pearson correlation coefficient; see Thioulouse et al., 2018, p. 185), ranging from 0 to 1, as a measure of global correlation between matrices, and tested its significance with a Monte Carlo permutation test using 999 permutations (Manly, 2007). The closer the RV is to 1, the greater the global similarity between the two matrices.

All multivariate analyses were performed on the individuals from both populations together. For the intrasite comparison in CH, we reran the analyses only on this population to then be able to compare the scores of the individuals according to the three areas of the site (Figure 1). Moreover, since we have 4 years of data (2016–2019), we checked for the effect of the year on our results. In terms of the relationship between immunological parameters and mineral elements, we had too much imbalance in the amount of data between years to make comparisons (*n*_2016_ = 42, *n*_2017_ = 29, *n*_2018_ = 10, *n*_2019_ = 24). However, we examined the relationship between parasite burden and mineral elements and found that the observed trend was the same between study years (see the relationship between individual scores on the first axis of the PCA on the parasite burden and the individual scores on the first axis of the PCA on the concentrations of mineral elements in Figure S2). As a result, we no longer took the year into account in the following results. Similarly, sex effects were tested, but no detectable differences were detected in mineral element concentrations except for copper (Student's *t* test: *n* = 233, *t* = −2.43, *p* value = .02) or in the scores of the individuals in each coinertia analysis. We thus pooled males and females together in each analysis. We then compared mineral element concentrations, parasite burdens, immunological parameters, and individual scores for each PCA/HSA/coinertia analysis between sexes (no effect) and study sites [Prediction 4a] using Student's t test and between the three areas of CH using the Kruskal–Wallis test [Prediction 4b] (nonparametric: unbalanced number of samples per area) and post hoc Dunn tests. Pearson's correlations were performed to verify the correlation between the covariates used in the coinertia analyses and to study the relationship between body mass and the score of individuals from each PCA/HSA/coinertia analysis [Prediction 3]. As coinertia assesses the global correlation between two sets of variables and suggests which variables from one set (e.g., mineral elements) are the most correlated with the variables of another set (e.g., immunology), we also calculated the correlation between these identified pairs of variables to assess their relationships more precisely using Pearson's correlations. We also studied the relationship between body mass and the score of individuals from each PCA/HSA/coinertia analysis [Prediction 3]. In roe deer, body mass is known to depend on the Julian date (Vanpé et al., 2007) and sex (Gaillard et al., 1996). We did not find any significant changes in body mass according to the Julian date (slope of the linear regression between body mass and Julian date ± SE = −0.017 ± 0.011, *t* = −1.46, *p* value = .15) or sex (Mann & Whitney test: *n* = 233, *W* = 6755, p value = 0.38). However, body mass was corrected for each study site by taking the residuals of the linear model [P3], as young roe deer are heavier at TF than at CH (Mann & Whitney test: *n* = 233, *W* = 2520, *p* value < .001). Finally, as the samples were collected between January and March at each study site, we tested for the effect of the Julian date on the data collected for each group of parameters by performing a linear model with individual scores on the first axis of the PCA for mineral elements and parasitic burden or on the first axis of the HSA for the immunological parameters as the response variables and the Julian date as a fixed effect (see Table S1B). The Julian date had a significant effect on the concentrations of three mineral elements in CH only: Ca (estimate ± SD, *p* value; 6.78 ± 3.06, .03), Co (0.0003 ± 0.001, 0.01), and Fe (0.70 ± 0.26, 0.01). Thus, we did not consider the effect of the Julian date on our results since the data were restricted to a few elements at a single study site.

## 3 RESULTS

### 3.1 Mineral element concentrations

**FIGURE 2:**
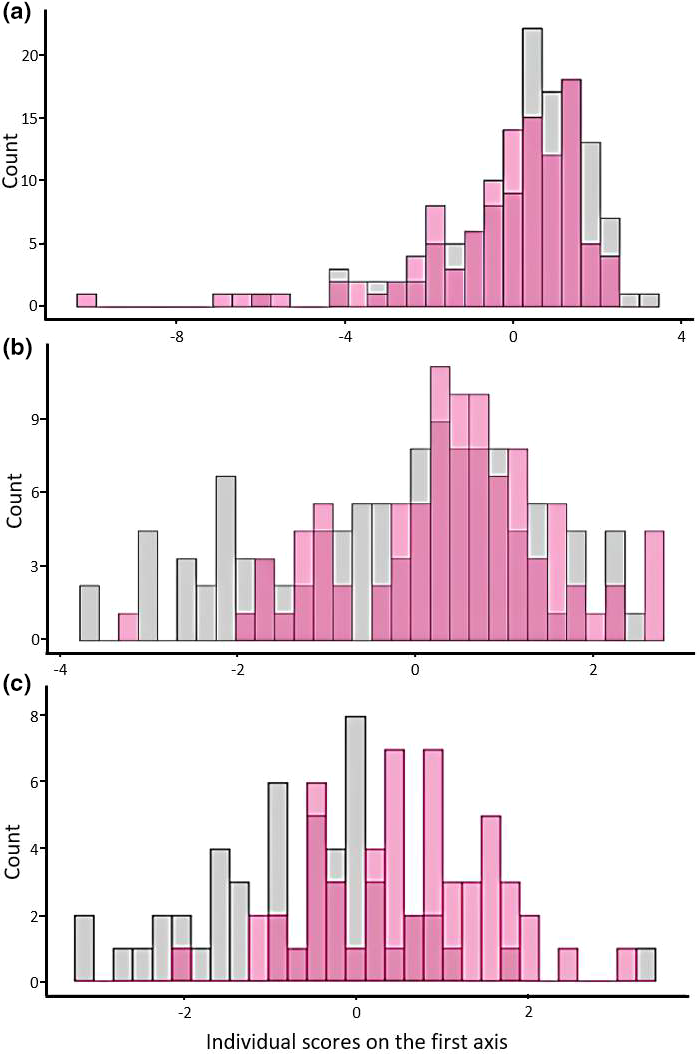
Scores of individuals on the first axis of the Principal Component Analysis or Hill & Smith Analysis on (a) mineral element concentrations, (b) parasite burden, (c) immunological parameters depicting the distribution of CH (in gray) and TF (in pink) scores on this axis.

The first principal component of the PCA on mineral element concentrations captured 32.06% of the total inertia and was much more important than all the following axes (see Figure S3a), leading us to retain only this axis for interpretation. The scores of individuals on the first axis showed that a few individuals were isolated on the right (Figure 2a). This raises the question of the effect of these individuals on the definition of the principal axis (i.e., the risk that a few individuals are driving the results of the analysis). However, when we performed the PCA without these individuals, we obtained the same structure of our variables, which showed that these isolated individuals did not influence the result of the PCA.

**FIGURE 3:**
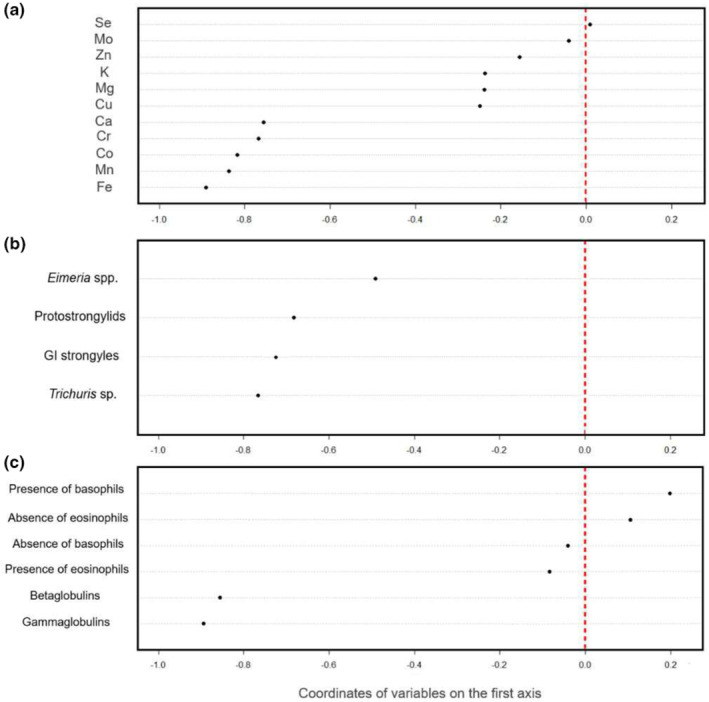
Coordinates of the variables on the first axis of the Principal Component Analysis or Hill & Smith Analysis on (a) mineral element concentrations, (b) parasite burden, (c) immunological parameters in two populations of roe deer in France. The red dotted vertical line represents the score of 0. These scores represent the Pearson's correlation coefficient between a variable and the first axes.

All the elements were positively correlated with the first axis of the PCA (Figure 3a), indicating a gradient in the data opposing animals with large concentrations of all the mineral elements (positive values of the first principal component, on the right of the red line) to animals with low concentrations of these elements (negative values of the first principal component, on the left of the red line). Individual scores on the first axis were negatively correlated with body mass (Pearson's correlation *R* = −0.15, df = 231, *p* value = .02), indicating that individuals with low body mass have more mineral elements in their hair. Moreover, although the distributions of scores of individuals from the two populations largely overlapped (Figure 2a), the Student's *t* test showed a difference between populations in the mean rank of individual scores on the first axis (*n* = 233, *t* = 2.47, *p* value = .01). Differences between sites were mainly explained by higher concentrations of Mg, Co, Mn, and Mo at TF and higher concentrations of Ca at CH (Table 1). Finally, when we compared the scores of individuals on the first axis of the PCA between the three areas of the CH, no detectable differences were found (Kruskal–Wallis test: *n*_1_ = 51, *n*_2_ = 26, *n*_3_ = 26; *H* = 0.46, df = 2, *p* value = .80).

**TABLE 1:** Comparison between the population (CH and TF) of the mineral element concentrations measured in roe deer hair samples (median, mean ± SD [min–max]; in μg/g of dry mass), the parasite burden (median, mean ± SD [min–max]; in egg per gram of feces for Gastrointestinal strongyles, *Trichuris* sp.; in oocysts per gram of feces for *Eimeria* spp. and in larvae per gram of feces for Protostrongylids), the concentration (median, mean ± SD [min–max]; in g/L) of immunological parameters (i.e., globulins).

		Trois‐Fontaines		Chize		Mann and Whitney	
		Median	Mean ± SD [Min–Max]	Median	Mean ± SD [Min–Max]	*W*	*p*‐Value
Mineral element concentrations (*n* = 233)	Calcium (Ca)	476.76	637.65 ± 724.95 [268.06–7285.35]	554.88	704.99 ± 598.36 [316.02–4889.71]	8226	**.03**
	Cobalt (Co)	0.048	0.072 ± 0.074 [0.019–0.53]	0.028	0.034 ± 0.021 [0.01–0.12]	3142	**<.001**
	Chromium (Cr)	0.16	0.24 ± 0.22 [0.004–1.28]	0.49	0.22 ± 0.20 [0.045–1.61]	6795.5	1
	Copper (Cu)	6.27	6.34 ± 0.92 [4.68–12.038]	6.49	6.66 ± 1.24 [5.16–17.28]	8087	.06
	Iron (Fe)	55.9	92.93 ± 138.03 [21.00–1245.51]	57.97	76.86 ± 51.87 [19.62–291]	7185	1
	Potassium (K)	4188.95	4402.76 ± 1192.3 [2215.99–8113.69]	4639.52	4838.35 ± 1694.23 [2218.78–13437.72]	7775	.25
	Magnesium (Mg)	269.84	314.74 ± 164.12 [80.70–1076.88]	202.08	262.27 ± 228.50 [60.47–1961.22]	4798	**<.001**
	Manganese (Mn)	5.3	9.16 ± 12.55 [1.91–84.35]	2.49	4.12 ± 6.84 [0.68–71.73]	2775.5	**<.001**
	Molybdenum (Mo)	0.046	0.18 ± 1.01 [0.017–10.53]	0.03	0.080 ± 0.37 [0.01–3.88]	3367	**<.001**
	Selenium (Se)	0.24	0.26 ± 0.11 [0.019–0.61]	0.22	0.28 ± 0.20 [0.068–1.34]	6027	.6
	Zinc (Zn)	72.14	76.54 ± 17.34 [44.55–137.90]	74.22	75.58 ± 13.16 [48.4–115.87]	6954	1
Parasite burdens (*n* = 190)	GI Strongyles	7.5	20.52 ± 28.56 [0–135]	15	31.60 ± 42.90 [0–270]	5161.5	.14
	*Trichuris* sp.	7.5	27.33 ± 76.02 [0–645]	30	124.81 ± 252.38 [0–2160]	6165	**<.001**
	*Eimeria* spp.	15	281.90 ± 1151.38 [0–10,125]	15	267.60 ± 810.52 [0–6855]	4447.5	.93
	Protostrongylids	1.3	19.76 ± 53.03 [0–248]	7	56.88 ± 155.16 [0–900]	5443.5	**.03**
Immunological parameters concentrations (*n* = 105)	Betaglobulins	5.3	5.41 ± 0.98 [3.5–9]	6.1	6.16 ± 1.36 [3.3–12.9]	1766	**<.001**
	Gammaglobulins	13.3	13.55 ± 2.98 [7.3–19.6]	17.4	18.66 ± 5.51 [9–34.9]	2015.5	**<.001**
	Basophils	0	0.08 ± 0.12 [0–0.51]	0	0.06 ± 0.10 [0–0.60]	1565.5	.59
	Eosinophils	0	0.02 ± 0.04 [0–0.19]	0	0.01 ± 0.02 [0–0.10]	1341.5	.56

*Note*: In bold: statistically significant differences in element concentration between the two populations. We implemented Holm's multiple testing correction procedure for the *p*‐value estimation (see Legendre and Legendre, 1998).

### 3.2 Relationship between parasite burden and mineral element concentrations

A clear break was observed in the decrease in the eigenvalues after the first component (Axis 1: 45.29% of total inertia; see Figure S3b) of the PCA on parasite burden; therefore, only the first axis was interpreted. This axis exhibited a clear pattern of correlation between all the parasites (Figure 3b), opposing animals with a high burden of all parasites (positive values of the first principal component, on the right of the red line) to animals with a low burden of parasites (negative values of the first principal component, on the right of the red line). Individual scores on the first axis were negatively correlated with body mass (Pearson's correlation *R* = −0.49, df = 188, *p* value < .001), indicating that individuals with low body mass have a greater parasite burden. Moreover, the CH‐related scores were greater than the TF‐related scores (Student's *t* test: *n* = 190, *t* = −3.80, *p* value < .001; see Figure 2b), and these differences were attributed to greater *Trichuris* sp. and protostrongylid burdens (Table 1). In CH (for a comparison of the element concentrations among the three areas, see Table S2), the differences in scores among the three areas were significant (Kruskal–Wallis test: *n* = 103, Khi^2^ = 6.93, df = 2, *p* value = .03). However, the Holm‐adjusted *p* values of the Dunn test were not significant. The pairs of areas that appeared to show the greatest differences in scores were Areas 1–2 (Dunn test: *n*_1_ = 51, *n*_2_ = 26, *Z* = −2.17, *p* value = .09) and Areas 1–3 (Dunn test: *n*_1_ = 51, *n*_3_ = 26, *Z* = −2.14, *p* value = .09).

We then performed a coinertia analysis on the two tables containing mineral element concentrations and parasite burden. There was a clear break in the decrease in the eigenvalues after the first axis (71.17% of total inertia), indicating that most of the coinertia was expressed on the first axis (see Figure S4a). We interpreted only this axis. The association between these two tables was statistically significant despite a low RV (RV = 0.044, *p* value = .02; many large arrows indicate a low correlation between the two tables in Figure S4b). Nevertheless, we could identify an association between these tables, as individuals with positive scores calculated from mineral element concentrations also exhibited, in general, positive scores calculated from parasite burdens.

**FIGURE 4:**
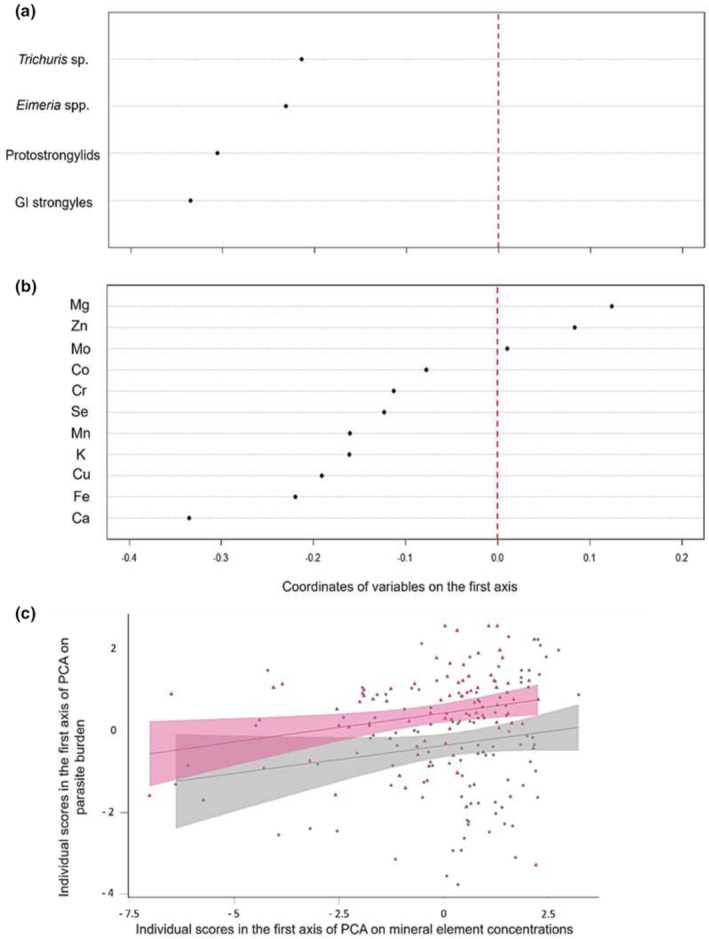
Co‐inertia analysis of the two tables containing mineral element concentrations and parasite burden, respectively. (a) Coordinates of the variables in the table containing parasite burden on the first axis of the co‐inertia analysis. (b) Coordinates of the variables in the table containing mineral element concentrations on the first axis of the co‐inertia analysis. (c) Relationship between the score of individuals on the first axis of the PCA on parasite burden and the score of individuals on the first axis of the PCA on mineral element concentrations according to population studied (in pink: TF with Pearson's correlation *R* = 0.26, df = 85, *p*‐value = .02); in gray: CH with Pearson's correlation *R* = 0.16, df = 101, *p*‐value = .11.

The first axis opposed individuals with high concentrations of most mineral elements (mainly Ca, Fe, Cu, K, and Mn, as indicated by higher scores on the first axis) and a high burden of parasites (mainly GI strongyles and protostrongylids, as indicated by higher scores on the first axis) to individuals with low concentrations of most mineral elements and low parasite burdens (Figure 4a,b). Thus, animals with the highest concentrations of most elements (except Mg, Zn, and Mo) were also the most parasitized (see Figure S5, which presents the global concentrations of mineral elements in individuals according to the burden of each parasite). This observed trend was the same for the two studied populations (Figure 4c) and between the four study years (see Figure S2). Nevertheless, there was a significant difference in the scores of individuals between TF and CH (student's *t* test: *n* = 190, *t* = −3.35, *p* value < .001), with higher scores at CH (i.e., reflecting a higher concentration of Ca and higher values of *Trichuris* sp. and protostrongylids at CH; see Table 1). Finally, body mass was significantly related to the scores of individuals on the first axis of the coinertia analysis (Pearson's correlation *R* = −0.48, df = 188, *p* value < .001), indicating that individuals with low body mass have higher mineral element concentrations and a greater parasite burden.

We could not identify any significant association between mineral element concentrations in roe deer hair and parasite burden in CH (low RV = 0.051, *p* value = .15; see comparison of the element concentrations and parasite burden between the three areas in Table S2). Therefore, we did not carry out an intrasite analysis to compare the different areas of the site.

### 3.3 Relationships between immunological parameters and mineral element concentrations

The first principal component of the HSA for immunological parameters captured 40.44% of the total inertia and was much more important than all the following axes (see Figure S3c), leading us to retain only this axis for interpretation. This axis was mainly represented by gamma‐globulins and beta‐globulins, which were positively correlated (Pearson's correlation *R* = 0.51, df = 103, *p* value < .001; see Figure 3c), indicating a gradient opposing animals with high quantities of globulins (i.e., gamma‐globulins and beta‐globulins) to animals with low quantities. Although the Pearson correlation coefficient was high (i.e., *R* =0 .51), we considered that there was no collinearity between the two globulins, in agreement with the findings of Dormann et al. (2013), who defined a collinearity threshold of *R* = 0.7. Individual scores on the first axis were negatively correlated with body mass (Pearson's correlation *R* = −0.22, df = 103, *p* value = .03), indicating that individuals with low body mass have higher globulin concentrations in their blood. Moreover, the individual scores on the first axis were greater at CH than at TF (student's *t* test: *n* = 105, *t* = −5.47, *p* value < .001; see Figure 2c); this difference is mostly explained by the greater concentrations of globulins at CH (Table 1). When we compared the scores among the areas at CH (see comparison of the globulin concentrations between the three areas in Table S2), we found no detectable difference (Kruskal–Wallis test: *n* = 51, *H* = 3.98, df = 2, *p* value = .14).

We then performed a coinertia analysis on the two tables containing mineral element concentrations and immunological parameters. There was a clear break in the decrease in the eigenvalues after the second axis (Axis 1: 53.85% of total inertia; Axis 2: 32.40% of total inertia; see Figure S6a). We thus interpreted the first two axes. The association between these two tables was statistically significant (RV = 0.069, *p* value = .025). The interpretation of the two first coinertia axes confirmed the low correlation between the two tables (i.e., many large arrows; see Figure S6b). Although the structure on the first two axes was much less clear than that in previous analyses, we could nevertheless identify a trend between mineral element concentrations and immunological concentrations.

**FIGURE 5:**
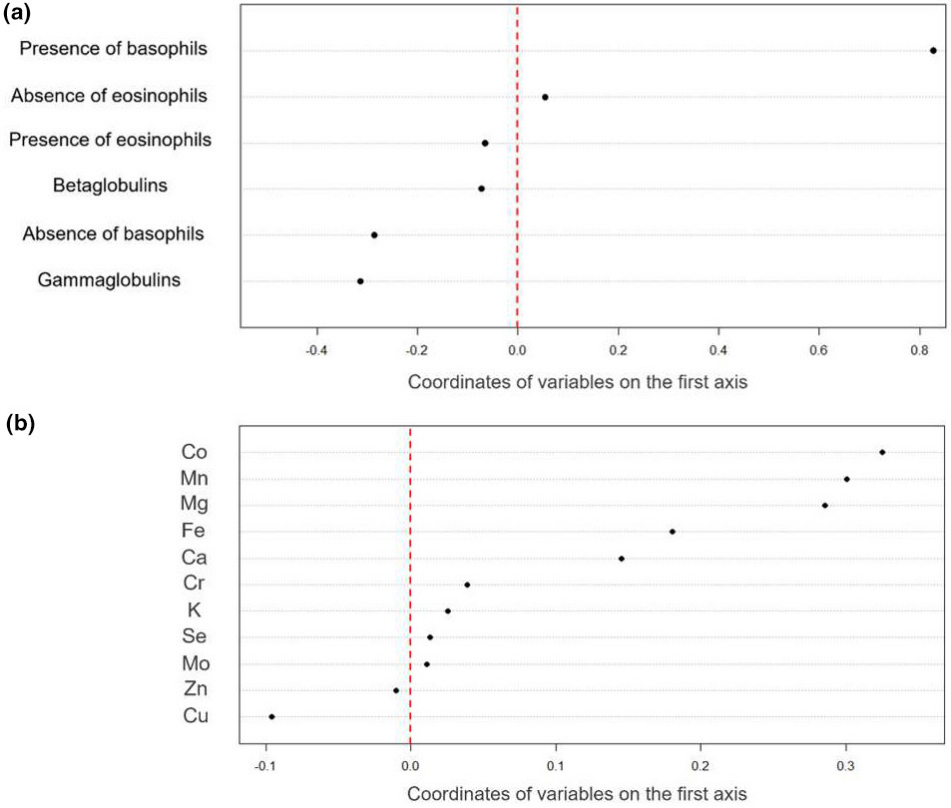
Co‐inertia analysis of the two tables containing mineral element concentrations and immunological parameters, respectively. Coordinates of the variables on the first axis of the co‐inertia analysis in the table containing (a) immunological parameters and (b) mineral element concentrations.

**FIGURE 6:**
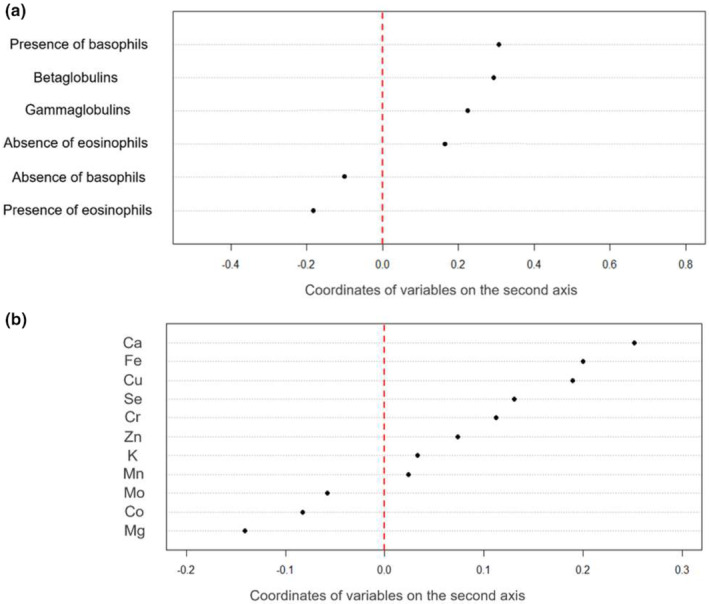
Co‐inertia analysis of the two tables containing mineral element concentrations and immunological parameters, respectively. Coordinates of the variables on the second axis of the co‐inertia analysis in the table containing (a) immunological parameters and (b) mineral element concentrations.

**FIGURE 7:**
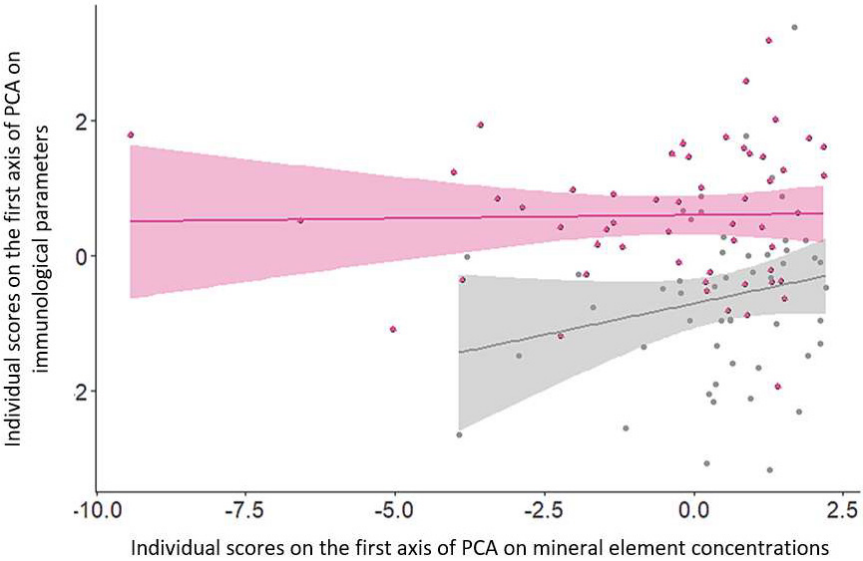
Relationship between the score of individuals on the first axis of the HSA on immunological parameters and the score of individuals on the first axis of the PCA on mineral element concentrations according to population studied. In pink: TF with Pearson's correlation *R* = 0.023, df = 52, *p*‐value = .87. In gray: CH with Pearson's correlation *R* = 0.21, df = 49, *p*‐value = .14.

The first axis opposed animals with high concentrations of most mineral elements (mainly Co, Mn, Mg, Fe, and Ca, visible by higher scores on the first axis) and the presence of basophils in their blood to animals with the opposite characteristics (Figure 5). The second axis opposed animals with high concentrations of many mineral elements (i.e., Ca, Fe, Cu, and, to a lesser extent, Se, Cr, and Zn), the presence of basophils, and high concentrations of globulins in their blood to animals with the opposite characteristics (Figure 6). The observed trend seemed more pronounced in CH (see Figure 7 for the relationship between individual scores on the first axis of PCA on immunological parameters and individual scores on the first axis of PCA on mineral element concentrations). Individual scores also differed according to the studied population (Student's *t* test: *n* = 105, *t* = 3.94, *p* value < .001 [Axis 1]; Axis 2: *n* = 105, *t* = −4.16, *p* value < .001 [Axis 2]). Finally, body mass was not significantly related to the scores of individuals on the first axis of the coinertia analysis (Pearson's correlation *R* = 0.13, df = 103, *p* value = .19), but it was related to the second axis (Pearson's correlation *R* = −0.20, df = 103, *p* value = .04), indicating that individuals with lower body mass have higher concentrations of some mineral elements (i.e., Ca, Fe, Cu, and, to a lesser extent, Se, Cr, and Zn) associated with higher concentrations of globulins.

## 4 DISCUSSION

The aim of the present study was to assess how winter parasite burden and immunological parameters were related to essential mineral element concentrations measured in hair as an indirect measure of the mineral status and the quality of the diet in autumn in young roe deer from two populations subjected to different ecological contexts. Based on our initial predictions, only one was verified (P2), with our results showing a positive relationship between immunological parameters (mainly the presence of basophils but also globulins) and mineral element concentrations (i.e., Co, Mn, Mg, Fe, Ca, and Cu and, to a lesser extent, Se, Cr, and Zn) in hair. For the others, we did not find the expected trends. Indeed, our results showed strong heterogeneity of individual phenotypes, with clear gradients opposing animals with low body mass associated with high concentrations of mineral elements (in particular, Ca, Fe, Cu, K, and Mn), high parasitic burden, and high globulin concentrations to animals with the opposite characteristics, not supporting the P3 prediction (i.e., body mass being associated positively with mineral element concentrations and immunological parameters and negatively with parasite burden). We also expected low concentrations of essential mineral elements to be associated with high parasite burdens (Koski & Scott, 2001; Suttle & Jones, 1989) and to play a role in immune defenses against endoparasites (e.g., roles of Co, Mo; McClure, 2008). In contrast to our first expectation (P1), parasitism, especially GI strongyle and protostrongylid burdens, was positively associated with the concentrations of mineral elements, particularly Ca, Fe, Cu, K, and Mn. This positive relationship between mineral element concentrations and parasite burdens was similar at both study sites, unlike the positive relationship between immunological parameters and mineral elements, which was only present at CH. Only low differences in mineral element status were observed between populations, as the distributions of individual scores for the PCA of mineral element concentrations largely overlapped. These differences included higher concentrations of Ca and lower concentrations of Mg, Co, Mn, and Mo in individuals from CH than in those from TF (P4a). No difference was found among the three CH areas (P4b). Therefore, our fourth hypothesis (i.e., P4, we expected to find lower mineral element concentrations and greater variability in phenotypic profiles in Chizé compared to Trois‐Fontaines [a], and inside the Chizé site [b]) was only partially supported due to some differences observed between Chizé and Trois‐Fontaines for certain elements but not all, and not inside the Chizé site. Although differences between males and females are known for mineral element status (Draghi et al., 2023; van Beest et al., 2023), we did not find any effect of sex on our mineral element concentrations (except for copper). This result could be attributed to the measurement period and the studied age class, that is, young individuals, which do not reflect the different specific needs of adult males and females, such as the development of antlers in males and gestation or lactation in females. For copper, whose concentrations are on average higher in males, an explanation could be that this element is involved in the synthesis of testosterone (Chang et al., 2011), which is then not found in females. However, among ungulates, no reference allows us to give a more concrete explanation.

A parasitic infection caused by nematodes or coccidia is known to impact the host's capacity to absorb nutrients and can lead to weight loss (Maublanc et al., 2009; Ramana, 2012). Moreover, parasites such as GI strongyles can lead to a reduction in food intake and the efficiency of different metabolic mechanisms, such as protein utilization in the host immune response (Coop & Holmes, 1996). Our observation of a greater parasite burden in young roe deer with a low body mass therefore seems to reflect these findings. However, in these individuals, the observation of higher mineral element concentrations is difficult to explain, given that they were rather expected to have lower mineral element concentrations (i.e., inappropriate concentrations to use for their immune defenses and/or a lower percentage of ingested elements reflecting their low body mass). Parasite infections can also induce behavioral changes in the host that may cause weight loss, meaning that not all parasite‐associated weight loss occurs through direct parasite interference with nutrient absorption. One explanation for our result could be that the high mineral element content reflects the host's careful selection of food resources that are richer in mineral elements. A host with a low body mass could indeed become more selective by targeting food that can minimize the risk of infection or that is rich in antiparasitic compounds (Kyriazakis et al., 1998). Although no studies have documented this phenomenon in wild ungulates, a study in sheep showed that individuals parasitized by nematodes chose a diet containing a greater proportion of nutrients (Cooper, 1996). Roe deer can feed on a wide range of foods (e.g., seeds, fruits, dicots, shrubs, and brambles), which each have different mineral element contents (Hocking & Pate, 1977), and is very flexible in its feeding behavior, particularly between seasons and type of habitat, according to the availability of feeding resources, physiological requirements, chemical composition of the plants (Barrere et al., 2022; Verheyden & Duncan, 1996), and certainly according to their health, as shown for sheep (Cooper, 1996). Although this has never been shown in this species to our knowledge, roe deer could select plants or parts of plants of different nutritional qualities (Tixier et al., 1997) depending on their parasitic status. Furthermore, the selection of foods richer in mineral elements has already been established in birds. Indeed, a study of insectivorous birds in Australia showed that the nutritional quality of insects plays an important role in food selection because, among the range of available prey, birds mainly choose those richest in mineral elements (i.e., containing high concentrations of Ca, Cu, Fe, K, Mg, Mn, and Zn; Razeng & Watson, 2015) to maximize energy intake (Kaspari & Joern, 1993; McCarty & Winkler, 1999) and specific nutrients for their metabolism (Graveland & Van Der Wal, 1996; Naef‐Daenzer et al., 2000). These elements could thus be important determinants of food choice, and this could also be the case for roe deer, particularly parasitized ones. Mineral elements such as Co, Cr, Cu, Fe, and Mn are likely to be involved in immunity directly or indirectly via their roles in other physiological systems that regulate immunity (Cerone et al., 1995; Larsen et al., 1988; Lequeux, 2016; McClure, 2008; Park et al., 2004), which may suggest the selection of foods richer in these elements to activate the immune system against parasites. This could explain why we observed higher concentrations of mineral elements in the parasitized young roe deer. It would be interesting to verify this finding by including diet data (i.e., mineral element content) or habitat use from roe deer to better understand the link between the use of resources by individuals and their mineral element profile.

We found associations between many mineral elements (i.e., Co, Mn, Mg, Fe, Ca, and Cu) and several immunological parameters, mainly basophils but also globulins (i.e., verifying the second prediction P2), in young roe deer with low body mass. Basophils are granulocytes that are rarely found in blood under homeostatic conditions. Nevertheless, the frequency of these cells increases in the blood, and they play important roles in type 2 immune responses, for example, when there is an allergic inflammation or an infection by helminths (Obata‐Ninomiya et al., 2020). As already mentioned, parasitic infections elicit a type 2 response and an increase in antibody (or immunoglobulin) levels, which can be followed by those of beta‐ and gamma‐globulin fractions. Therefore, in our study, young roe deer, which have a low body mass and a high parasite burden, probably exhibited an adaptive immune reaction (Gilot‐Fromont et al., 2012). The positive correlation between the concentrations of immunological parameters and the concentrations of mineral elements (i.e., Ca, Cu, Fe, K, Mg, Mn, and Se) may thus reflect the essential role of these elements in the development of optimal immune responses during parasitic infections (Aboshady et al., 2020; Lee et al., 2011; Stear et al., 2003; Strain et al., 2002). Indeed, as a reminder, they function in the maintenance of physical barriers (skin and mucous membranes), cell differentiation and proliferation, inflammation, antioxidant protection (i.e. protein secretion), and, more generally, the regulation or triggering of immune responses (Hughes & Kelly, 2006; McClure, 2008; Paul & Dey, 2015). For instance, magnesium deficiency could be associated with inadequate immune modulation (Kubenam, 1994), and selenium deficiencies could lead to a loss of immune competence (Powolny et al., 2023; Rivera et al., 2003; Spallholz et al., 1990).

Moreover, this positive relationship between mineral element concentrations and immunological parameters in young roe deer was found mainly at CH (i.e., greater slope in Figure 7) relative to TF. However, a positive relationship between mineral element concentrations and parasite burdens was found at both study sites, despite the marked differences in environmental conditions. Overall, at CH, as at TF, individuals with low body mass exhibiting the highest mineral element concentrations also displayed the highest parasite burdens. This is surprising because the environments the two populations inhabit differ in several parameters, such as the quantity and quality of resources or the soil composition. Some of these differences are reflected in the concentrations of some mineral elements, with more Mg, Co, Mn, and Mo (i.e., microelements) at TF and more Ca at CH. It is also surprising that no difference is visible within the CH site, but this is probably due to the amount of data that are not balanced between the sites. The forest at TF is highly productive, with abundant and high‐quality forage, whereas at CH, the forest is limited by low resource availability (Gaudry et al., 2018; Pettorelli et al., 2006). Forest productivity is known to be strongly dependent on soil quality, including mineral element composition (Ranger, 2018). Even though the soil is calcareous at both sites, it is deeper and more fertile at TF than at CH, where it is stonier and shallower and where limestone patches prevent roots from growing deeper into the ground. A soil is most fertile when it has nutrients, for example, mineral elements, in sufficient quantities. This could explain why there were more mineral elements at this study site. However, at present, we do not have data verifying that environmental conditions (i.e., soil composition and food resources) influence mineral element concentrations in roe deer hair. Furthermore, on the second axis of co‐inertia between immunological parameters and essential elements, it seems that calcium is the element most correlated with globulins and the presence of basophils. This would explain why this relationship is mainly visible at Chizé, the study site where there is more calcium. In immune cells, intracellular Ca can regulate many cellular functions, including cytokine production and cell proliferation (Lippolis, 2011). We can therefore assume that in Chizé, where calcium is more present in roe deer hair but also probably in the environment, this element is the one found most involved in metabolism, with in particular an involvement in immune function. Regarding the positive relationship between parasitic status and mineral status in roe deer, it seems that the environment in which individuals live does not influence this trend and that other factors at the individual level seem to be involved.

Therefore, further investigations are still needed to better understand these complex interactions. Most of the literature that has focused on dietary deficiencies and their effects on animal health comes from veterinary research and relates to livestock (Coop & Holmes, 1996; Droke & Spears, 1993; McClure, 2008; Parkins & Holmes, 1989). In wildlife, it is much more difficult to properly assess a species' requirement for essential mineral elements and to determine the threshold at which an individual can be considered to suffer from a deficiency. Studying wild populations is challenging because of the limited access to these animals and the limited opportunities to follow them for long periods (Wilson & McMahon, 2006). Therefore, our study provides novel information suggesting that a high parasite burden could predispose young roe deer with low body mass to have higher metabolic requirements associated with immunity, possibly leading to increased mineral element intake through increased food selection (i.e., rich in mineral elements). In addition, our study showed that an immune reaction, possibly following an infection, that is, high concentrations of basophils and globulins, is related to high concentrations of mineral elements, which may reflect the importance of these essential elements in the mobilization of the immune system.

However, our results are correlative and do not prove causality. We are aware that each element has a different function in the body, and not all elements can be linked in the same way to immune function or defense against parasites (McClure, 2008). For example, Cu deficiency can cause clinical problems in deer, namely enzootic ataxia and osteochondrosis (Grace & Wilson, 2002). Each essential element seems to have its own mode of action at the metabolic level, but there is still little evidence of these actions, particularly in wildlife. Moreover, the mineral elements can be related between them (Cygan‐Szczegielniak et al., 2018; Herrada et al., 2024). For instance, some studies have shown that the presence of Cu can influence the levels of Zn in the organism (Bremner & Beattie, 1995; López‐Alonso et al., 2005). There appears to be a metabolically significant antagonism between Zn and Cu, where the elements compete for absorption from the digestive tract (Bremner & Beattie, 1995). A recent study (Herrada et al., 2024) has notably shown that in roe deer, the concentration of certain mineral elements in the hair seems to be correlated, but these relationships were also looked at the population level. Furthermore, this study provided for the first‐time reference intervals for 22 elements in roe deer, including those studied here, considering therefore that most roe deer with element concentrations found in these reference intervals have «normal» concentrations of these elements in their organism. Given that essential elements are necessary to ensure optimal functioning of the body, we could imagine that roe deer not within these intervals would have inadequate concentrations in their organisms, with possible metabolic consequences. It would be interesting to see if, on a finer scale, these individuals outside the norms are sick, as has been observed for selenium deficiency, which can cause enzootic ataxia and osteochondrosis (Grace & Wilson, 2002).

Cygan‐Szczegielniak (2021) reported significant correlations between the concentrations of both mineral elements and toxic metals measured in different organs (i.e., muscle, liver, and kidneys) and in the hair of red deer (*Cervus elaphus*). For instance, hair concentrations were correlated with renal concentrations for Fe, Mg, Mn, and Cu, and hair concentrations were correlated with hepatic concentrations for Cd and Pb. However, the associations were positive in some cases (i.e., Fe, Mn, and Cu) but negative in others (i.e., Na, Ca, Mg, Pb, and Cd) (Cygan‐Szczegielniak, 2021). Conversely, in wood mice (*Apodemus sylvaticus*) sampled along a soil metal pollution gradient, positive correlations have been detected between Cd and Pb concentrations in hair and in the liver and/or kidneys, although the associations were weak for Cd and stronger for Pb (Tête et al., 2014). In another study conducted on wood mice sampled along the same pollution gradient, Powolny et al. (2019) reported positive relationships between concentrations in blood and in the liver and/or kidneys for Cd, Fe, Mo, Pb, Tl, and Zn, while no significant correlations were detected for Cu or Ti (Powolny et al., 2019). Cygan‐Szczegielniak (2021) stressed that accumulation in a specific tissue or organ may be linked to an increase or a decrease in the elemental concentrations in another part of the body, but such processes are influenced by the complex mechanisms of toxicokinetics and toxicodynamics (e.g., variable elimination rates in different tissues, metal binding to organic or inorganic molecules), age, sex, and body condition, and temporal variation in the uptake of elements from environmental sources (Powolny et al., 2019). In this study, we assumed that the use of hair to measure essential element concentrations represents the mineral status of individuals in autumn (Katz & Chatt, 1994). Monitoring the condition of animals can be difficult due to temporal variation in the absorption, transport, and storage of trace elements in different tissues of an organism (Bremner et al., 1981), particularly when these parameters must be linked to others that vary over different periods of time, that is, body condition (long term), parasite burden, or immune function (short term). A recent study on two species of deer, fallow deer and red deer, clearly showed that the status of some mineral elements (Cu, Se, Zn) is tissue‐specific (van Beest et al., 2023). Consequently, a better understanding of the relationships between essential elements and parasitism/immunity in roe deer will need to consider matrices other than hair in future studies. Specific studies on diet quality are also needed, and measuring all variables at the same time is necessary to confirm our results, as is considering possible annual differences, which may reflect the effect of climate, for example. Finally, in the study by Draghi et al. (2023), age‐related patterns of trace element accumulation were found. Potential age‐ and even sex‐related differences in mineral element concentrations should not be discarded, and it would be interesting to conduct the same study in older animals, which have different physiological needs (e.g., production of secondary sexual characteristics in males or pregnancy/lactation in females), to determine whether similar trends occur. Taking all these elements into account would make it possible to improve understanding of the direct and indirect effects of essential elements on short‐ or long‐term health indicators in wild populations.

## AUTHOR CONTRIBUTIONS

**Léa Bariod:** Conceptualization (equal); formal analysis (lead); methodology (equal); writing – original draft (lead). **Sonia Saïd:** Conceptualization (equal); funding acquisition (equal); methodology (equal); supervision (equal); writing – review and editing (equal). **Clément Calenge:** Formal analysis (supporting); writing – review and editing (equal). **Renaud Scheifler:** Methodology (equal); writing – review and editing (equal). **Clémentine Fritsch:** Writing – review and editing (equal). **Carole Peroz:** Writing – review and editing (equal). **Slimania Benabed:** Investigation (equal). **Hervé Bidault:** Investigation (equal). **Stéphane Chabot:** Investigation (equal). **François Débias:** Investigation (equal). **Jeanne Duhayer:** Investigation (equal). **Sylvia Pardonnet:** Investigation (equal). **Marie‐Thérèse Poirel:** Investigation (equal). **Paul Revelli:** Investigation (equal). **Pauline Vuarin:** Funding acquisition (equal); methodology (equal); writing – review and editing (equal). **Gilles Bourgoin:** Conceptualization (equal); funding acquisition (equal); methodology (equal); supervision (equal); writing – review and editing (equal).

## FUNDING INFORMATION

This work was also supported by the VetAgro Sup, and the ‘Office Français de la Biodiversite’ (OFB) under partnership agreement CNV20.0943. This work was carried out thanks to the financial support of the IDEXLYON Project of the University of Lyon within the framework of the “Investment for the Future” Program (ANR‐16‐IDEX‐0005).

## CONFLICT OF INTEREST STATEMENT

The authors declare no conflict of interest.

## Supporting information


Appendix S1

## Data Availability

The data that support the findings of this study are openly available in Dryad at https://doi.org/10.5061/dryad.k3j9kd5fj.
