# Supplementary material for: Essential mineral elements in roe deer: Associations with parasites and immune phenotypes in two contrasting populations
Source: Ecol Evol. 2024 Oct 29;14(10):e11613. doi: 10.1002/ece3.11613 (PMC11519704; doi:10.1002/ece3.11613)
Supplement: Supplementary file 1 — Appendix S1 [file ECE3-14-e11613-s001.docx]

**Essential mineral elements in roe deer: Associations with parasites and immune phenotypes in two contrasting populations**

Léa Bariod^*^, Sonia Saïd, Clément Calenge, Renaud Scheifler, Clémentine Fritsch, Carole Peroz, Slimania Benabed, Hervé Bidault, Stéphane Chabot, François Débias, Jeanne Duhayer, Sylvia Pardonnet, Marie-Thérèse Poirel, Paul Rivelli, Pauline Vuarin, Gilles Bourgoin

*Corresponding author

E-mail: [bariod.lea@orange.fr](mailto:bariod.lea@orange.fr)

# SUPPORTING INFORMATION

**Table S1**: **A**) Distribution of sampling according to month and year by study site and **B**) relationships (LM) between the Julian date and the score of individuals on the first axis of the PCA for the mineral elements and the parasitic burden, or on the first axis of the HSA for the immunological parameters. In bold: statistically significant p-value.

**A**)

|  |  | **Trois-Fontaines** | | | **Chizé** | | |
| --- | --- | --- | --- | --- | --- | --- | --- |
|  |  | **Hair samples** | **Feces samples** | **Blood samples** | **Hair samples** | **Feces samples** | **Blood samples** |
| **2016** | **January** | 7 | 7 | 7 | 8 | 7 | 8 |
|  | **February** | 17 | 16 | 15 | 8 | 3 | 7 |
|  | **March** | 0 | 0 | 0 | 5 | 5 | 5 |
| **2017** | **January** | 10 | 7 | 6 | 21 | 20 | 7 |
|  | **February** | 25 | 18 | 12 | 16 | 14 | 2 |
|  | **March** | 0 | 0 | 0 | 3 | 3 | 2 |
| **2018** | **January** | 8 | 8 | 0 | 8 | 7 | 2 |
|  | **February** | 14 | 12 | 0 | 12 | 11 | 6 |
|  | **March** | 0 | 0 | 0 | 13 | 7 | 2 |
| **2019** | **January** | 8 | 5 | 3 | 2 | 2 | 0 |
|  | **February** | 22 | 16 | 11 | 19 | 16 | 6 |
|  | **March** | 0 | 0 | 0 | 7 | 6 | 4 |

**B**)

|  | Individual scores | Julian date effect | |
| --- | --- | --- | --- |
|  |  | Estimate ± SD | P-value |
| Trois-Fontaines | Mineral elements | -0.02 ± 0.01 | 0.08 |
|  | Parasite burdens | 0.003 ± 0.008 | 0.97 |
|  | Immunological parameters | -0.0007 ± 0.01 | 0.94 |
| Chizé | Mineral elements | -0.02 ± 0.008 | **0.003** |
|  | Parasite burdens | -0.01 ± 0.008 | 0.13 |
|  | Immunological parameters | -0.02 ± 0.008 | 0.48 |

**
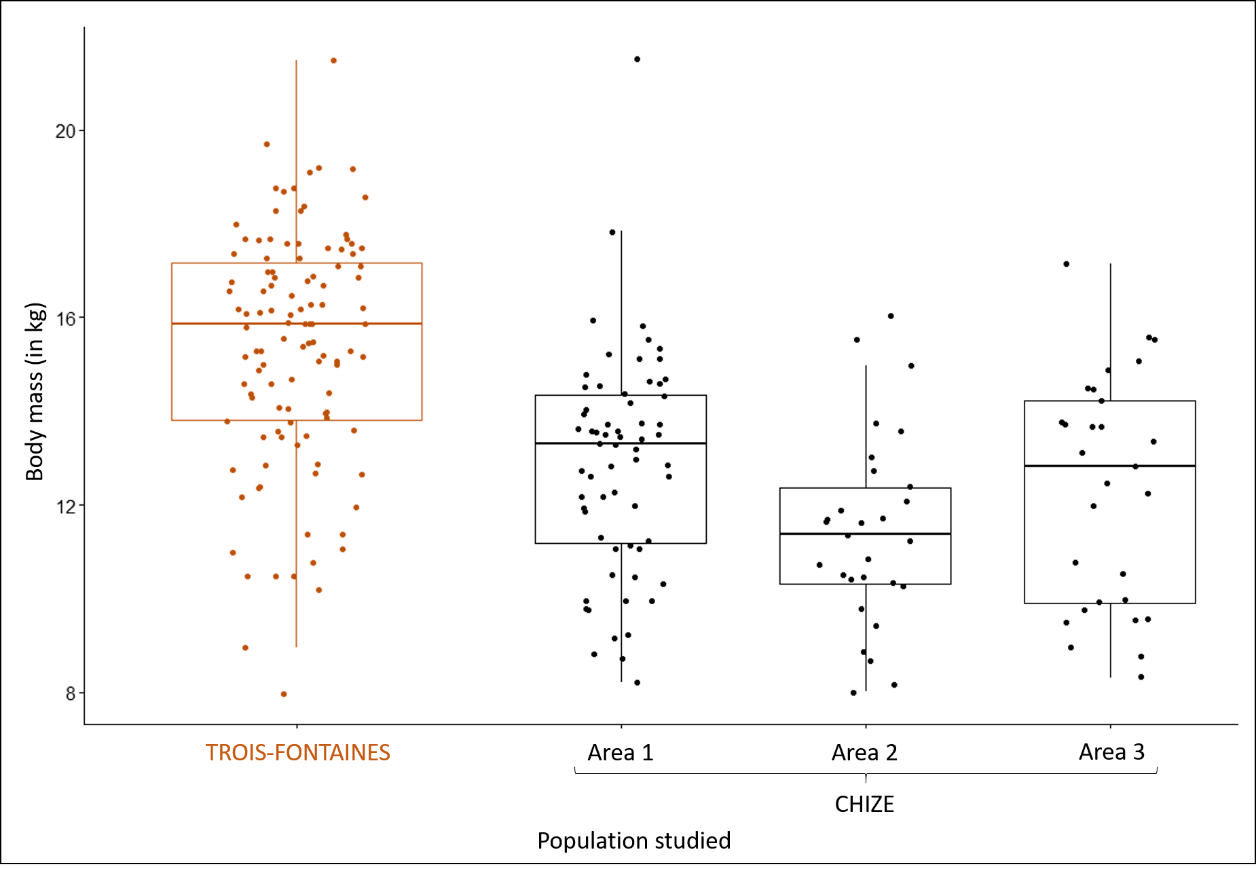
**

**Figure S1**: Body mass of the roe deer according to the study site (Trois-Fontaines in orange, n = 111; Chizé in black, n = 122) and the areas of Chizé (Area 1, n = 64; Area 2, n = 29; Area 3, n = 29). The horizontal line in the box corresponds to the median.

**
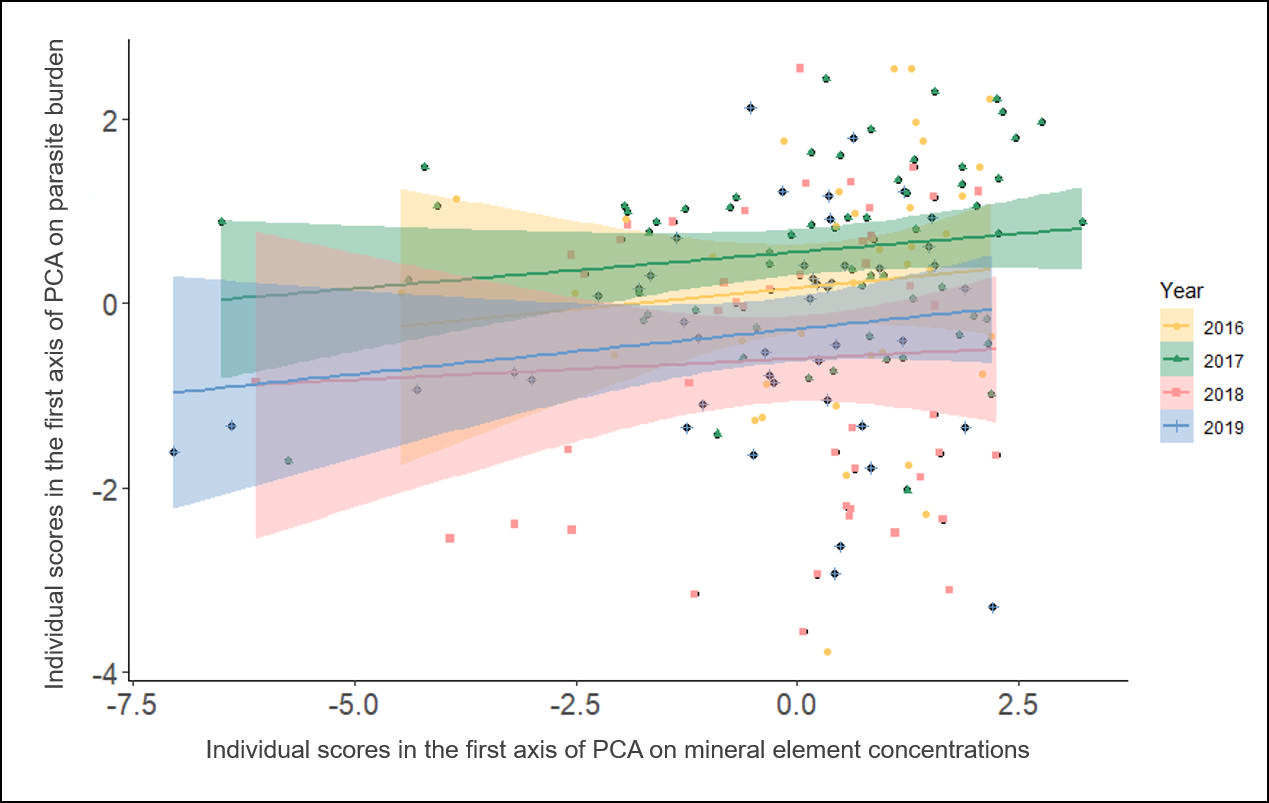
**

**Figure S2**: Relationship between the score of individuals on the first axis of the PCA on parasite burden and the score of individuals on the first axis of the PCA on mineral element concentrations according to the study year (in yellow: 2016 with Pearson’s correlation R = 0.11, df = 36, p-value = 0.53; in green: 2017 with Pearson’s correlation R = 0.16, df = 60, p-value = 0.20; in red: 2018 with Pearson’s correlation R = 0.03, df = 43, p-value = 0.84; in blue: 2019 with Pearson’s correlation R = 0.16, df = 43, p-value = 0.28).

**
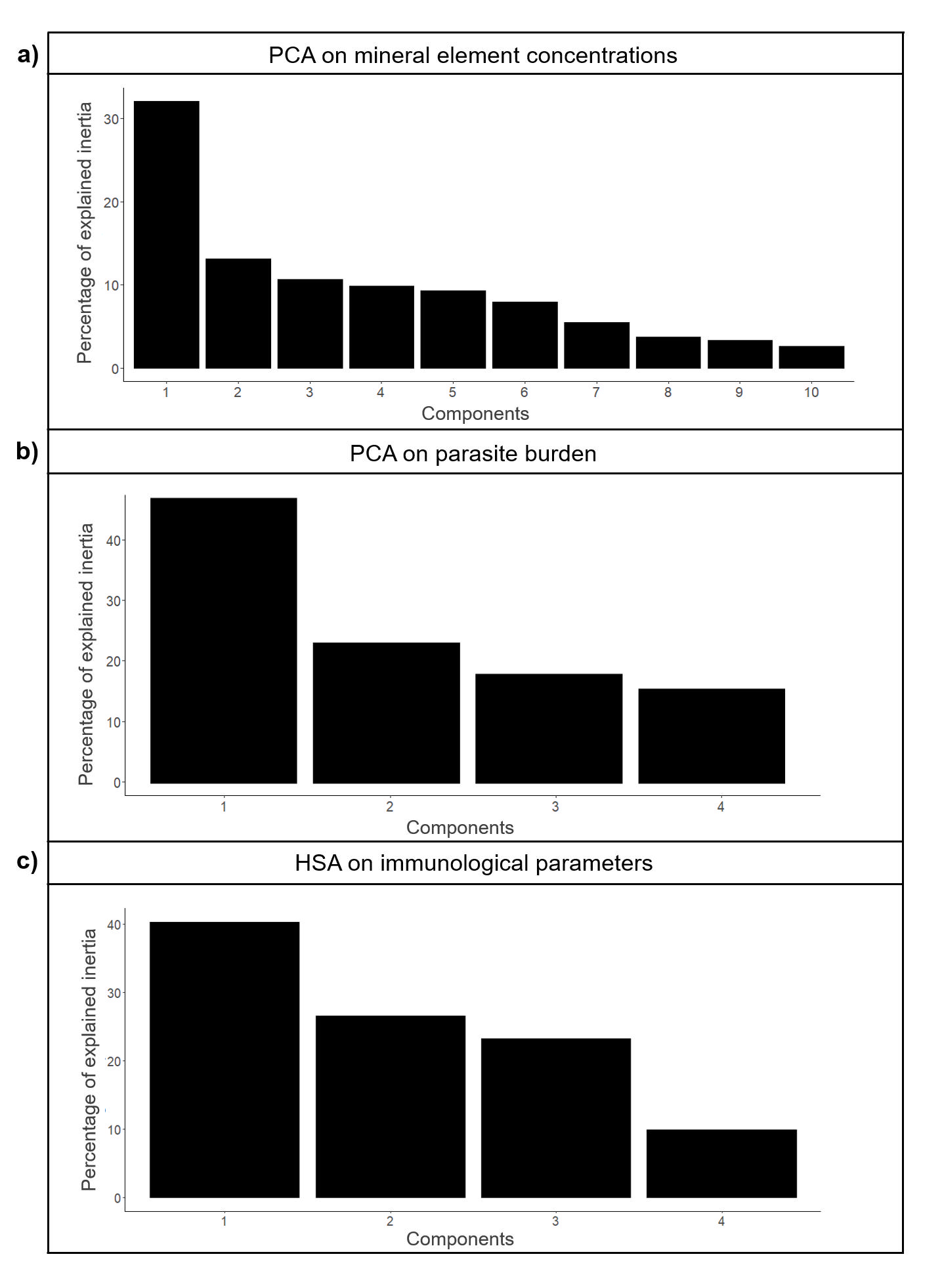
Figure S3**: Decomposition of the variance among principal components of the Principal Component Analysis or the Hill & Smith Analysis on **(a)** mineral element concentrations, **(b)** parasite burden, **(c)** immunological parameters, expressed as the percentage of inertia explained by each component.

**Table S2:** Comparison between the three areas in Chizé of (**A**) the mineral element concentrations (median, mean +/- sd [min-max]; in μg/g of dry mass) measured in roe deer hair samples (n[Area1] = 64, n[Area2] = 29, n[Area 3] = 29), (**B**) the parasite burden (median, mean +/- sd [min-max]; in egg per gram of feces for Gastrointestinal strongyles, *Trichuris* sp.; in oocysts per gram of feces for *Eimeria* spp. and in larvae per gram of feces for Protostrongylids; n[Area1] = 51, n[Area2] = 26, n[Area 3] = 26), (**C**) the concentration (median, mean +/- sd [min-max]; in g/L) of immunological parameters (n[Area1] = 27, n[Area2] = 8, n[Area 3] = 16). In bold: statistically significant differences in element concentration between the two populations. We implemented the Holm’s multiple testing correction procedure for the p-values estimation (see Legendre and Legendre, 1998).

|  | |  |  | | | | | |  | Kruskal-Wallis | | | | | Dunn's test | | | | | |
| --- | --- | --- | --- | --- | --- | --- | --- | --- | --- | --- | --- | --- | --- | --- | --- | --- | --- | --- | --- | --- |
|  | |  | Area 1 | | Area 2 | | Area 3 | |  |  |  |  |  |  | 1-2 | | 1-3 | | 2-3 | |
|  | |  | Median | Mean ± sd [Min-Max] | Median | Mean ± sd [Min-Max] | Median | Mean ± sd [Min-Max] |  | *Khi²* | | *P-value* | | | *P-value* | | | | | |
| MINERAL ELEMENT CONCENTRATIONS | Calcium (Ca) | | 554.88 | 692.31 ± 556.19 [337.23-4562.76] | 521.45 | 808.06 ± 852.18 [316.02-4889.71] | 557.12 | 629.92 ± 328.33 [320.17-1964.54] |  | | 0.66 | | 0.72 | - | | | | | |  |
|  | Cobalt (Co) | | 0.03 | 0.04 ± 0.02 [0.01-0.12] | 0.03 | 0.03 ± 0.02 [0.01-0.11] | 0.02 | 0.03 ± 0.02 [0.01-0.07] |  | | 2.79 | | 0.25 | - | | | | | |  |
|  | Chromium (Cr) | | 0.15 | 0.2 ± 0.15 [0.04-0.79] | 0.18 | 0.18 ± 0.09 [0.06-0.39] | 0.17 | 0.29 ± 0.32 [0.06-1.61] |  | | 1.04 | | 0.59 | - | | | | | |  |
|  | Copper (Cu) | | 6.55 | 6.52 ± 0.75 [5.16-9.26] | 6.54 | 6.63 ± 0.63 [5.4-8.16] | 6.27 | 6.99 ± 2.21 [5.49-17.28] |  | | 0.65 | | 0.72 | - | | | | | |  |
|  | Iron (Fe) | | 58.4 | 80.96 ± 57.77 [25-291] | 57.13 | 69.19 ± 44.12 [27.9-241.62] | 56 | 75.50 ± 45.50 [19.62-182.5] |  | | 0.41 | | 0.82 | - | | | | | |  |
|  | Potassium (K) | | 4667.79 | 4741.30 ± 1452.78 [2218.78-11201.21] | 5563.25 | 5625.14 ± 2134.96 [2390.94-13437.72] | 3897.54 | 4265.76 ± 1447.51 [2458.62-10246.87] |  | | 11.93 | | **0.003** | 0.06 | | 0.16 | | **0.003** | |  |
|  | Magnesium (Mg) | | 192.25 | 248.92 ± 184.02 [60.47-918.13] | 198.16 | 319.68 ± 356 [79.13-1961.22] | 206.39 | 234.33 ± 134.46 [62.74-564.26] |  | | 0.77 | | 0.68 | - | | | | | |  |
|  | Manganese (Mn) | | 2.55 | 3.66 ± 3.19 [0.68-17.03] | 2.78 | 5.88 ± 12.94 [0.81-71.73] | 2.32 | 3.38 ± 2.70 [0.91-12.36] |  | | 0.47 | | 0.79 | - | | | | | |  |
|  | Molybdenum (Mo) | | 0.03 | 0.03 ± 0.01 [0.01-0.08] | 0.03 | 0.04 ± 0.02 [0.02-0.12] | 0.05 | 0.23 ± 0.76 [0.02-3.88] |  | | 19.19 | | **< 0.001** | 0.07 | | **< 0.001** | | 0.18 | |  |
|  | Selenium (Se) | | 0.16 | 0.22 ± 0.15 [0.07-0.71] | 0.27 | 0.30 ± 0.19 [0.09-0.087] | 0.36 | 0.38 ± 0.26 [0.08-1.34] |  | | 13.85 | | **< 0.001** | 0.07 | | **0.002** | | 0.5 | |  |
|  | Zinc (Zn) | | 74.07 | 74.67 ± 11.76 [52.08-110.13] | 77.95 | 79.43 ± 13.25 [56.38-104.94] | 72.21 | 73.74 ± 15.53 [48.4-115.87] |  | | 3.39 | | 0.18 | - | | | | | |  |
| PARASITE BURDENS | GI Strongyles | | 7.5 | 26.62 ± 44.83 [0-270] | 30 | 39.81 ± 42.78 [0-135] | 30 | 33.17 ± 39.18 [0-165] |  | | 4.20 | | 0.12 | - | | | | | |  |
|  | *Trichuris* sp. | | 15 | 80.74 ± 137.29 [0-480] | 52.5 | 202.21 ± 440.41 [0-2160] | 120 | 133.85 ± 133.92 [0-465] |  | | 5.10 | | 0.08 | - | | | | | |  |
|  | *Eimeria* spp. | | 7.5 | 303.68 ± 1052.72 [0-6855] | 75 | 174.52 ± 293.24 [0-1185] | 22.5 | 289.90 ± 604.78 [0-2385] |  | | 3.10 | | 0.21 | - | | | | | |  |
|  | Protostrongylids | | 3.33 | 50.38 ± 159.28 [0-900] | 19.25 | 72.21 ± 174.42 [0-673.6] | 7 | 54.29 ± 129.31 [0-613.3] |  | | 3.18 | | 0.20 | - | | | | | |  |
| IMMUNOLOGICAL PARAMETERS CONCENTRATIONS | Betaglobulins | | 6.2 | 6.28 ± 0.7 [5.2-8.3] | 6 | 6.1 4± 1.06 [5-8.5] | 5.5 | 5.97 ± 2.19 [3.3-12.9] |  | | 6.15 | | 0.05 | - | | | | | |  |
|  | Gammaglobulins | | 19.40 | 20.26 +- 6.01 [11.2-34.9] | 15.65 | 16.28 ± 2.99 [12-21.8] | 16.1 | 17.15 ± 4.91 [9-25.6] |  | | 4.58 | | 0.1 | - | | | | | |  |
|  | Basophils | | 0 | 0.01 ± 0.03 [0-0.1] | 0 | 0.01 ± 0.03 [0-0.06] | 0 | 0.01 ± 0.02 [0-0.06] |  | | 2.8 | | 0.25 | - | | | | | |  |
|  | Eosinophils | | 0.05 | 0.05 ± 0.06 [0-0.21] | 0.08 | 0.15 ± 0.21 [0-0.6] | 0 | 0.04 ± 0.08 [0-0.27] |  | | 0.17 | | 0.92 | - | | | | | |  |

**
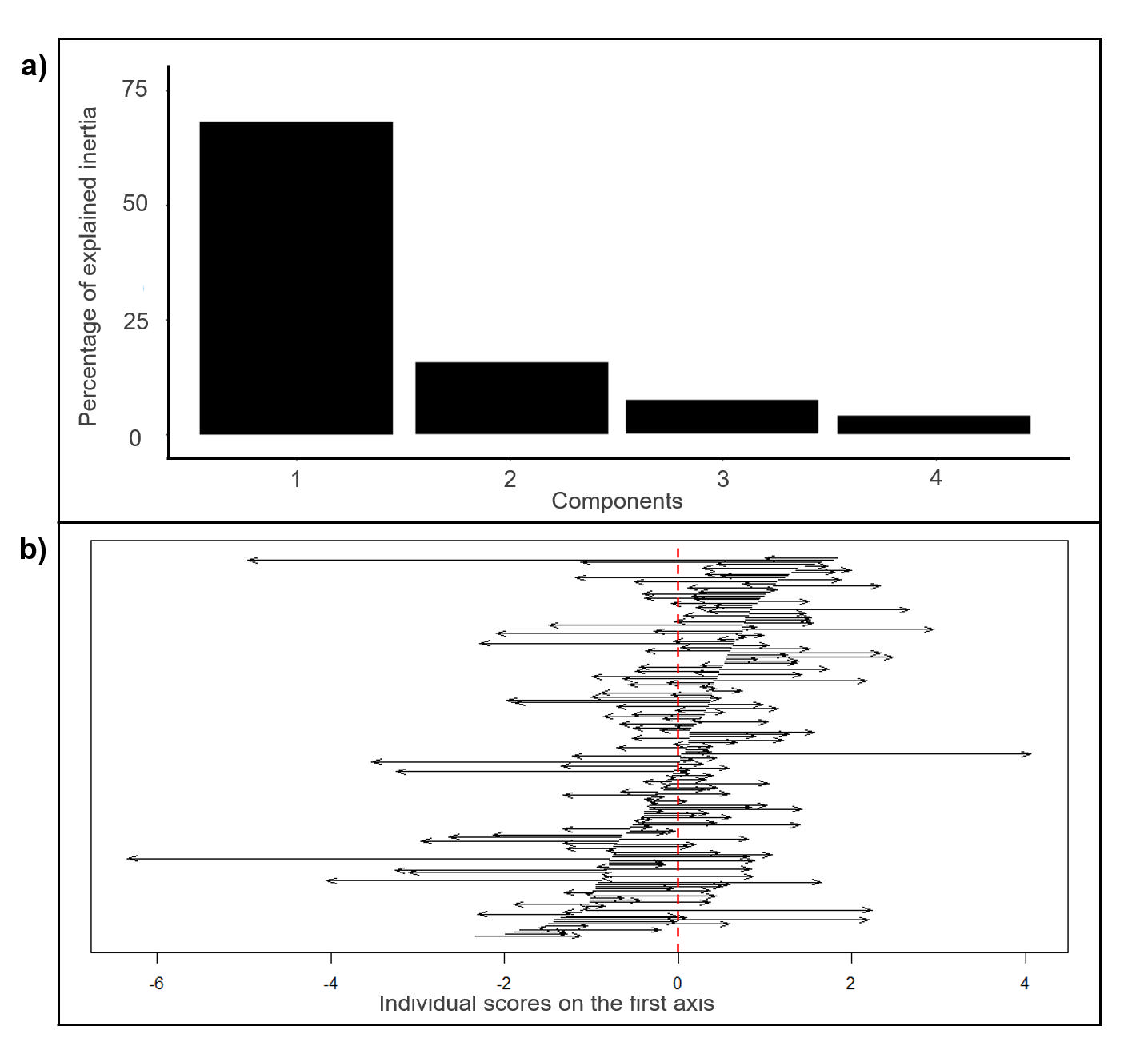
**

**Figure S4:** Co-inertia analysis of the two tables containing mineral element concentrations and parasite burden, respectively. (**a**) Eigenvalues diagram of the analysis; the eigenvalues are presented as the percentage of co-inertia explained by each axis (first axis selected for interpretation). (**b**) Individual scores from smallest to largest in the co-inertia plan: mineral element concentration (origin of arrows) and corresponding linear combination of parasite burden data (extremity of arrows). As the co-inertia analysis finds two linear combinations of the variables of the two tables that are characterized by the largest covariance, a high correlation between the two tables is characterized by a short arrow (i.e. many individuals having a similar score calculated from the two tables).

**
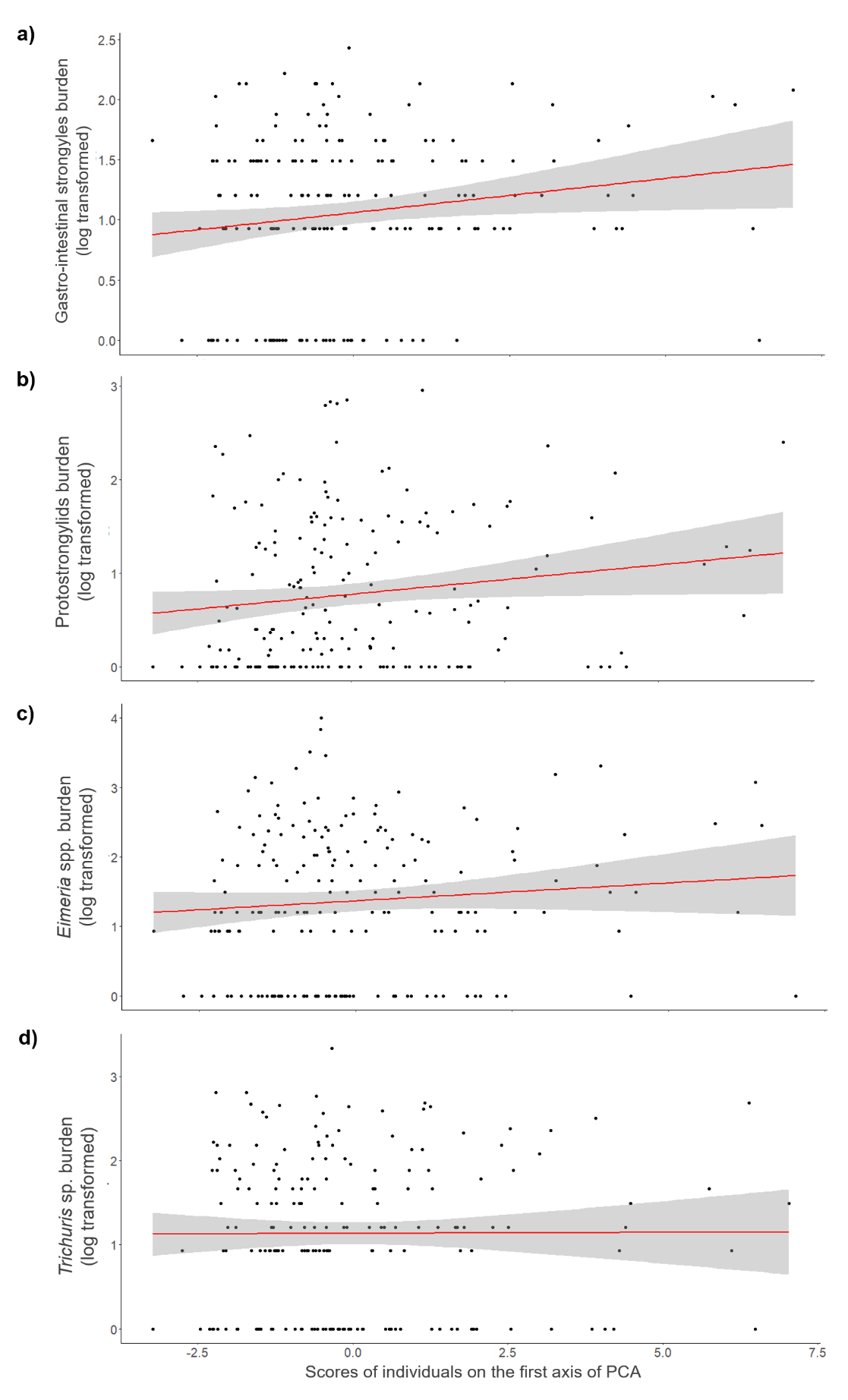
**

**Figure S5:** Relationship between the score of individuals on the first axis of the Principal Component Analysis on mineral elements and parasite burden, respectively, according to each parasite studied: (**a**) Gastrointestinal strongyles (Pearson’s correlation R = 0.16, df = 188, p-value = 0.03), (b) Protostrongylids (Pearson’s correlation R = 0.15, df = 188, p-value = 0.04), (c) *Eimeria* spp. (Pearson’s correlation R = 0.09, df = 188, p-value = 0.19), (d) *Trichuris* sp. (Pearson’s correlation R = 0.001, df = 188, p-value = 0.99).


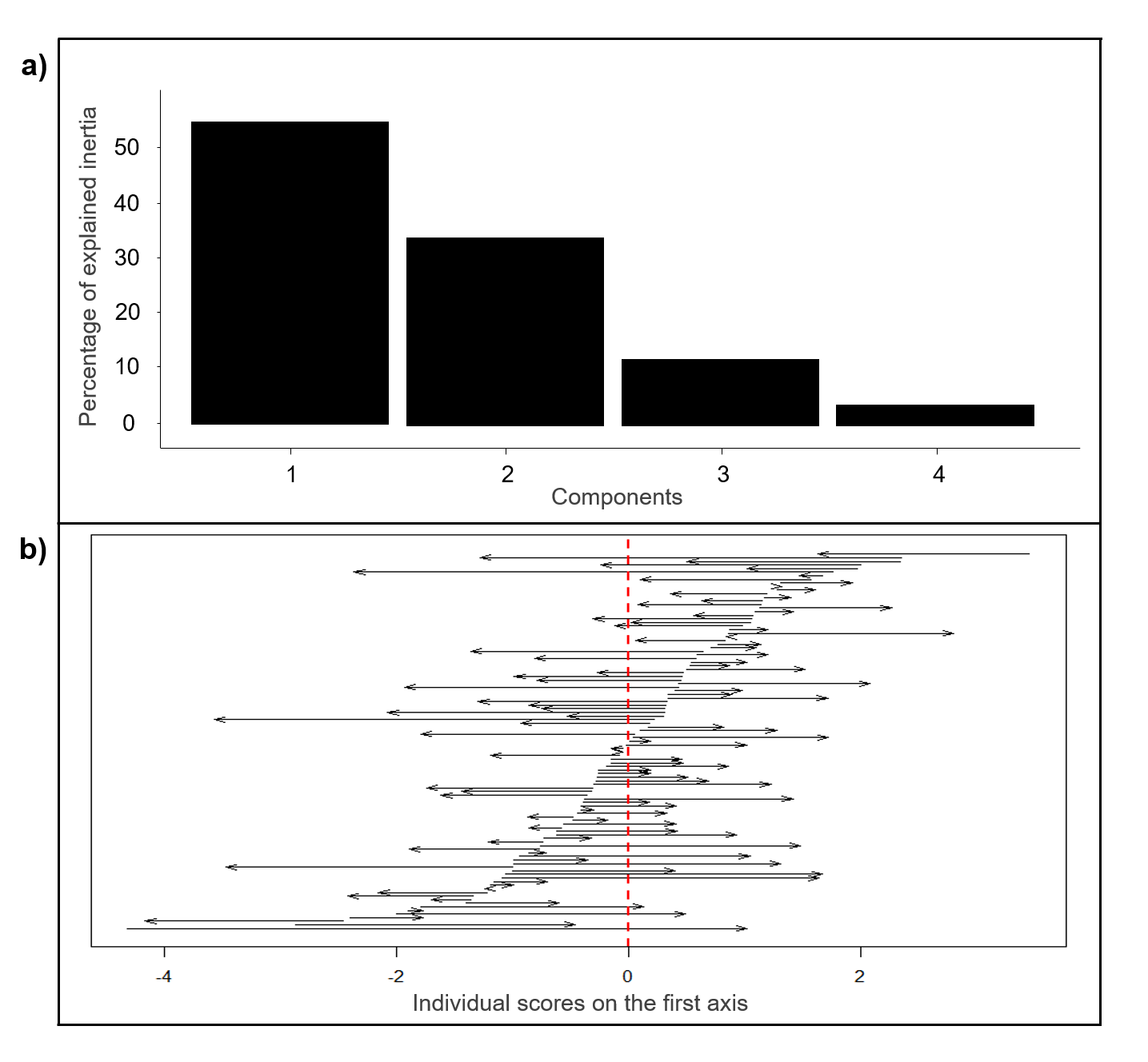


**Figure S6**: Co-inertia analysis of the two tables containing mineral element concentrations and immunological parameters, respectively. **(a)** Eigenvalues diagram of the analysis; the eigenvalues are presented as the percentage of co-inertia explained by each axis (two first axes selected for interpretation). **(b)** Individual scores from smallest to largest in the co-inertia plan: mineral element concentration (origin of arrows) and corresponding linear combination of parasite burden data (extremity of arrows). As the co-inertia analysis finds two linear combinations of the variables of the two tables that are characterized by the largest covariance, a high correlation between the two tables is characterized by a short arrow (i.e. many individuals having a similar score calculated from the two tables).
